# CpG binding protein (CFP1) occupies open chromatin regions of active genes, including enhancers and non-CpG islands

**DOI:** 10.1186/s13072-018-0230-0

**Published:** 2018-10-06

**Authors:** Louie N. van de Lagemaat, Maria Flenley, Magnus D. Lynch, David Garrick, Simon R. Tomlinson, Kamil R. Kranc, Douglas Vernimmen

**Affiliations:** 1 The Roslin Institute and Royal (Dick) School of Veterinary Studies, University of Edinburgh, Easter Bush, Midlothian, EH25 9RG UK; 2 MRC Molecular Haematology Unit, Weatherall Institute for Molecular Medicine, John Radcliffe Hospital, University of Oxford, Oxford, OX3 9DS UK; 3 Centre for Stem Cells and Regenerative Medicine, 28th Floor Guy’s Tower, Great Maze Pond, London, SE1 9RT UK; 4 INSERM, UMRS-1126, Institut Universitaire d’Hématologie, Université Paris Diderot, 75010 Paris, France; 5 MRC Centre for Regenerative Medicine, University of Edinburgh, 5 Little France Drive, Edinburgh, EH16 4UU UK; 6 Laboratory of Haematopoietic Stem Cell & Leukaemia Biology, Centre for Haemato-Oncology, Barts Cancer Institute, Queen Mary University of London, Charterhouse Square, London, EC1M 6BQ UK; 7 st John’s institute of dermatology, Great Maze Pond, London, SE1 9RT UK

**Keywords:** CFP1, CpG islands, Trithorax group proteins, Epigenetics, Enhancers

## Abstract

**Background:**

The mechanism by which protein complexes interact to regulate the deposition of post-translational modifications of histones remains poorly understood. This is particularly important at regulatory regions, such as CpG islands (CGIs), which are known to recruit Trithorax (TrxG) and Polycomb group proteins. The CxxC zinc finger protein 1 (CFP1, also known as CGBP) is a subunit of the TrxG SET1 protein complex, a major catalyst of trimethylation of H3K4 (H3K4me3).

**Results:**

Here, we used ChIP followed by high-throughput sequencing (ChIP-seq) to analyse genomic occupancy of CFP1 in two human haematopoietic cell types. We demonstrate that CFP1 occupies CGIs associated with active transcription start sites (TSSs), and is mutually exclusive with H3K27 trimethylation (H3K27me3), a marker of polycomb repressive complex 2. Strikingly, rather than being restricted to active CGI TSSs, CFP1 also occupies a substantial fraction of active non-CGI TSSs and enhancers of transcribed genes. However, relative to other TrxG subunits, CFP1 was specialised to TSSs. Finally, we found enrichment of CpG-containing DNA motifs in CFP1 peaks at CGI promoters.

**Conclusions:**

We found that CFP1 is not solely recruited to CpG islands as it was originally defined, but also other regions including non-CpG island promoters and enhancers.

**Electronic supplementary material:**

The online version of this article (10.1186/s13072-018-0230-0) contains supplementary material, which is available to authorized users.

## Background

In vertebrate genomes, CpG dinucleotides are relatively depleted, except in specific DNA regions with a high density of this dinucleotide. These regions are known as CpG islands (CGIs) and consist of short (~ 1000 bp) interspersed CpG-rich and predominantly unmethylated DNA sequences [1], which are associated with transcriptionally permissive chromatin state [2]. Interestingly, the human genome harbours roughly twofold more annotated CGIs than the mouse genome (27,000 versus 15,000) [3]. CGIs were originally identified in promoters of housekeeping genes and associated with H3K4me3 independent of gene activity [1]. However, it is now recognised that CGIs are also found in about half the promoters of developmentally regulated/tissue-specific genes [4]. In this group of CGIs, repressive polycomb group (PcG) complexes act to block transcription in inappropriate lineages or at non-expressing stages during the differentiation programme. It has been hypothesised that CGIs at developmentally regulated genes of higher organisms may be relics of ancestral CGIs which have been differentially maintained during evolution, for example, in humans and rodents [3–8]. Thus, at present, the functional significance of these CGIs for the correct regulation of developmentally regulated genes is poorly understood. A good example of this is the well-studied α-globin locus. Mouse α-globin genes, which lack an annotated CGI, exhibit tightly regulated tissue and developmental stage-specific regulation similar to that observed for the human α-globin genes, which contain a CGI and are regulated by PcG [9].

Several proteins have been described in mammals that bind specifically to CGIs. These proteins contain the CxxC zinc finger (ZF-CxxC) domain and include CFP1, MLL1, MLL2, KDM2A, KDM2B, TET1 and TET3 [10]. Whilst MLL1/2 also have inherent histone lysine N-methyltransferase activity, CFP1 does not, instead forming complexes with SET1A and SET1B methyltransferases, TrxG subunits of the SET1 family [11]. CFP1-SET1A/B complexes have been implicated in development [11, 12] and are known to target a proportion of H3K4 methylation to CGIs [12, 13]. CFP1 binds unmethylated CpG-rich sequence [14], and is associated with H3K4 trimethylation at transcription start sites (TSSs) irrespective of transcription level [15, 16], although recent evidence showed that it preferentially binds CGIs of actively transcribed genes [17]. Two other TrxG complexes, MLL3 and MLL4, lack ZF-CxxC domains [10] and have been suggested to be involved in deposition of H3K4me1 at enhancer elements [18–20].

Multiple phenotypes have been documented for CFP1 deficiency, both at the cellular and organismal level. CFP1 is required for development [21], differentiation of embryonic stem (ES) cells [22] and haematopoiesis [23–25]. CFP1 binds to unmethylated CGIs in mouse ES cells [15, 17] and mouse brain tissues [16], and knockout and knockdown of CFP1 in mouse ES cells and NIH3T3 fibroblasts result in reduction of H3K4me3 levels at CGIs [16, 17]. Mouse ES cells lacking CFP1 are viable but are unable to differentiate [22, 26]. In these cells, H3K4me3 was found to be mainly reduced at highly expressed genes, while the levels at other CGIs such as bivalent domains were largely unchanged [15]. These observations may be explained by the above-mentioned CFP1 preference for actively transcribed genes and the finding that MLL2 (rather than SET1) complexes are largely responsible for initial deposition of H3K4me3 at bivalent domains [27, 28].

Here we have analysed the genome-wide occupancy of CFP1 in two divergent human haematopoietic cell types: erythroid (ERY) and EBV-transformed B-lymphoid (EBV) cells. Previous studies using chromatin immunoprecipitation (ChIP) have been restricted to a couple of available antibodies with moderate signal, which consequently limited the sensitivity of the analysis [15, 29]. Here, using a non-commercial, high-affinity antibody [30], our analysis reveals that the presence of CFP1 at accessible CGI TSSs is associated with Pol II binding and gene expression. Moreover, H3K27me3, which is a mark of PcG (PRC2) activity, and binding of CFP1 are mutually exclusive at TSSs. Unexpectedly, CFP1 is also recruited to a substantial fraction of non-CGI-associated TSSs and active enhancer elements. In comparison with other TrxG complex subunits, CFP1 is specialised to active TSSs. Finally, our analysis revealed enrichment of CpG dinucleotide-containing motifs in CFP1 peaks at CGIs.

## Results

### CFP1 primarily binds at active CGI TSSs and is associated with transcription

We previously reported an association of CFP1 binding with transcription at the α-globin locus, using a mouse model in which the mouse α-globin locus was replaced by the human locus [29, 31]. In this “humanised” mouse model, the epigenetic regulation of the human locus in mouse erythroblasts mirrors that observed in human primary erythroblasts, including recruitment of PcG when the gene is silenced [29]. In these humanised mouse erythroid cells, the targeted deletion of a key human α-globin enhancer (MCS-R2, also known as HS-40) results in a strong reduction in α-globin transcription [31, 32]. This is associated with the maintenance of PcG binding [29] and an impairment of CFP1 recruitment to the human α-globin promoter [29]. Similarly, in human primary erythroid and lymphoid (EBV-transformed) cell types, where this locus is active and inactive, respectively, CFP1 is recruited to the CGI spanning the α-globin gene only in erythroid cells [29]. Experiments with a robust, non-commercial CFP1 antibody [30] confirmed these results in primary cells (Additional file 1: Fig. S1A) and in the humanised mouse model (Additional file 1: Fig. S1B). ChIP signals derived from this antibody were several fold stronger than a comparable commercial CFP1 antibody (Additional file 1: Fig. S1C). The non-commercial antibody was tenfold more sensitive (14,872 peaks, as described below, versus 1434 in erythroid cells), and in 1102 locations where the peaks overlapped it showed a median 2.3 fold higher enrichment of reads in peaks given a similar number of ChIP-seq reads and the same input data. Given this tenfold greater sensitivity and 2.3-fold greater specificity, it was the primary antibody used in this study.

**Fig. 1:**
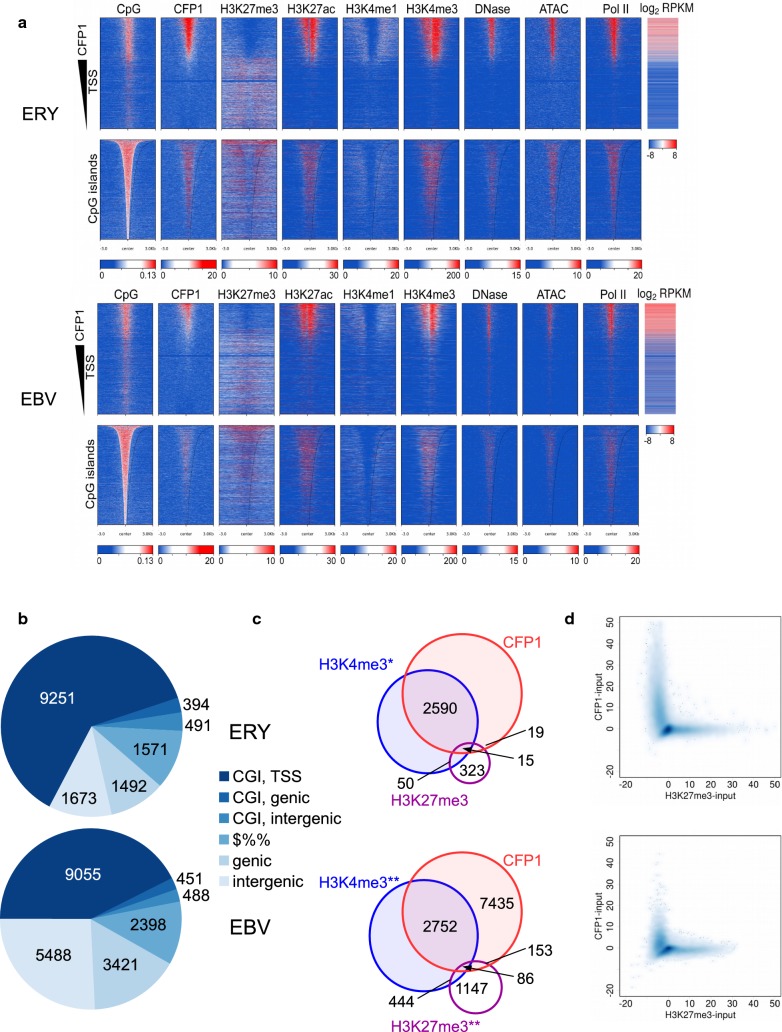
Genomic distribution of CFP1 relative to CpG islands, transcription start sites (TSSs) and marks of active and repressed chromatin. **a** Heatmap plots of human genomic 6-kb regions centred on TSSs (top) and CGIs (bottom) in erythroblasts (ERY) and Epstein-Barr virus transformed lymphoblast (EBV) cells. TSSs sorted in order of CFP1 coverage; CGIs sorted in order of size. Sequencing read depths shown for CFP1, H3K27me3, H3K27ac, H3K4me1, H3K4me3, DNase-seq, ATAC-seq and Pol II are net coverage after normalisation to 1x genome-wide and subtraction of an input data set similarly normalised. Gene expression (log_2_ RPKM) is shown to the right for TSSs in both cell types. **b** Location of CFP1 summits allocated in order of CGI TSS (< 1 kb), CGI genic, CGI intergenic, (non-CGI) TSS (< 1 kb), (non-CGI) genic and (non-CGI) intergenic. ERY, top; and EBV, bottom. **c** Venn diagrams showing CFP1 peaks within 1-kb of TSSs are strongly associated with H3K4me3 histone mark and poorly associated with H3K27me3 repressive histone mark. Cell types are ERY (upper) and EBV (lower). Public data sets: * NCBI GEO GSE36985, ** NCBI GEO GSE50893. **d** Mutual exclusivity of CFP1 and the H3K27me3 mark in ERY (upper) and EBV (lower) cell types

To identify genomic regions recruiting CFP1, we performed ChIP-seq in primary human lymphoid and erythroid cell types (biological replicates from Fig. S1A, Additional file 1). As shown in Fig. 1a, heatmaps of genomic TSSs and CGIs showed that these regions were strongly enriched in CFP1 binding. As shown in Fig. 1b, genomic CFP1 localisation was remarkably similar in both cell types, with a large number of peaks located at TSSs with annotated CGIs: 62.2% (9251/14,872) and 42.5% (9055/21,301) of genome-wide CFP1 summits were associated with CGI promoters in erythroid and lymphoid cells, respectively. Amongst non-CGI genomic regions of CFP1 binding, TSSs were less prominent (Fig. 1b). As shown in Fig. S1D (Additional file 1), CGI and non-CGI TSSs differed strongly in CpG content within a 1-kb window centred on TSSs. CpG content of representative CGI and non-CGI loci considered hereafter are graphed in Fig. S1D (Additional file 1) .

As shown in Fig. 1a, CFP1-bound TSSs were depleted in H3K27me3 and H3K4me1 histone marks, whilst being strongly enriched in H3K4me3. The association between CFP1 and H3K4me3 could either reflect deposition of H3K4me3 by CFP1-SET1A/B complexes, or binding to pre-existing H3K4me3, deposited by other TrxG complexes [33], through the PHD finger domain of CFP1 [17]. Accordingly, we found strong association between peaks of CFP1 and H3K4me3 in both erythroid and lymphoid cell types, both qualitatively at the level of heatmaps (Fig. 1a) and quantitatively in peak overlaps (Fig. 1c). Indeed, the fraction of CFP1 peaks overlapped with H3K4me3 was nearly 100% when the alternate peak finder, MACS2, was used (Additional file 1: Fig. S2).

In contrast to H3K4me3 and CFP1, little if any colocalisation was observed between CFP1 and H3K27me3. To further characterise depletion of H3K27me3 (a histone mark associated with PcG binding) in CFP1-bound regions, we plotted averaged read depth in a 2-kb window surrounding TSSs for CFP1 versus H3K27me3. As shown in Fig. 1d, TSSs showed mutual exclusivity between H3K27me3 and CFP1 occupancy, with marked preference for binding of either CFP1 or PRC2 (H3K27me3), or neither, but not both; this was observed consistently in erythroid and lymphoid cell types. This result demonstrates that mutual exclusivity of CFP1 and the H3K27me3 mark at TSSs, which was first described in the α-globin locus [29], is a general genome-wide mechanism in disparate cell types.

### Binding of CFP1 to housekeeping and tissue-specific gene TSSs

**Fig. 2:**
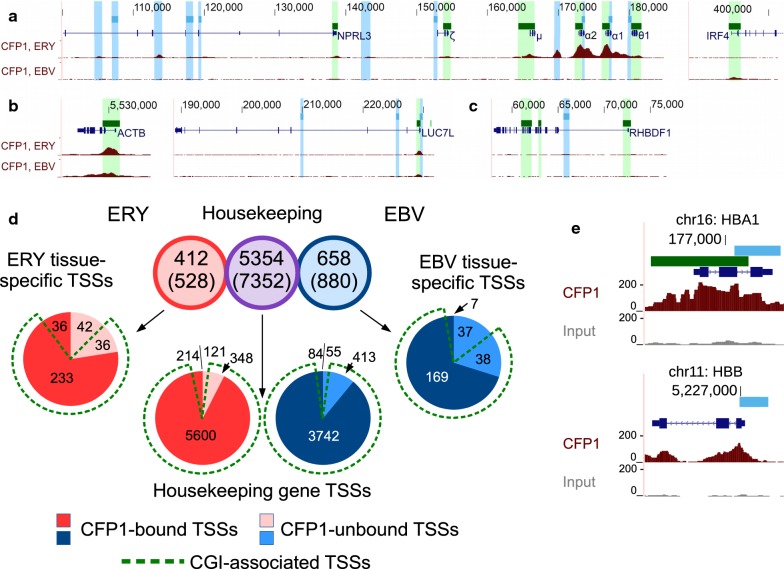
CFP1 localisation in TSSs of housekeeping and tissue-specific genes in ERY and EBV cell types. **a**–**c** CFP1 binding specifically to TSSs in ERY (upper) and EBV (lower) cell types. Chromosomal position specified in the human hg38 genome build. Pileups are shown scaled to 1x genome coverage, with full scale 0–200x depth. **a** Tissue-specifically expressed loci: α-globin (chr16, left, expressed in ERY) and IRF4 (chr6, right, expressed in EBV). **b** Housekeeping genes: ACTB (chr7, left) and LUC7L (chr16, right). **c** RHBDF1 locus (chr16) with CGI promoter, but not transcribed in ERY or EBV. **d** Upper circles show counts of tissue-specific ERY-expressed (left), housekeeping (centre) and tissue-specific EBV lymphoid-expressed (right) genes, with total TSSs shown in parentheses. Lower pie charts show analysis of CFP1 ChIP peaks computed in accessible TSSs of ERY (red) and EBV (blue) cell types. Darker colours represent TSSs with CFP1 peaks, and dashed green lines indicate TSSs having a CGI. **e** CFP1 at two loci expressed in ERY: α-globin gene HBA1, expressed from a CGI TSS, compared to β-globin gene HBB, expressed from a non-CGI TSS

We next assessed CFP1 occupancy at TSSs by gene class, specifically housekeeping genes and developmentally regulated/tissue-specific genes (Fig. 2 and Additional file 1: Fig. S3). Both the α-globin locus in erythroblasts (Fig. 2a, left) and IRF4 locus in lymphoid cells (Fig. 2a, right) exhibit lineage-specific binding of CFP1. Housekeeping genes such as ACTB and LUC7L (located just downstream of the α-globin locus) showed binding in both cell types (Fig. 2b). The CGI promoter of RHBDF1, which is not expressed in either cell type, was marked by H3K27me3 in both erythroid and lymphoid cell types (Additional file 1: Fig. S3C), but was not bound by CFP1 (Fig. 2c).

We then extended our analysis to genome-wide sets of housekeeping and developmentally regulated/tissue-specific genes. First, we used the Illumina Body Map RNA-seq data set of 16 human tissues to identify 5354 genes with similar expression across most or all of these tissues and termed these housekeeping genes (Fig. 2d; see “Methods”). Another 7150 genes with low expression in nine or more of 16 tissues were termed candidate tissue-specific genes. RNA-seq data sets from erythroblasts and EBV-transformed lymphoid cells were then used to identify candidate tissue-specific genes with elevated expression in these cell types (412 genes in erythroid and 658 in lymphoid cells; see “Methods”). Interestingly, CXXC1 mRNA, which encodes CFP1 protein, was denominated a housekeeping gene, with mean RPKM (reads per kilobase of transcript per million mapped reads) of 5.8 (± 3.1 SD). Public expression data sets for EBV and ERY cells showed overexpression of CXXC1 in EBV (RPKM = 50.6) and reduced expression in ERY (RPKM = 0.62). This mRNA expression difference corresponded to approximately twofold higher expression at the protein level in lymphoid cells (Additional file 1: Fig. S4).

To address the confounding effect of alternate TSSs of transcribed genes, some with or without CGIs, and some transcribed or not, we next limited our analysis to putatively expressed TSSs. These were identified by accessible chromatin, which was defined by the presence of 1x-normalised/input-subtracted ATAC (Assay for Transposase-Accessible Chromatin) signal > 10 within 1 kb of the TSS. Most putatively expressed TSSs regardless of gene class were marked by CGIs (defined by a distance < 1 kb; green dotted lines, Fig. 2d). However, a larger proportion of housekeeping gene TSSs were marked by a CGI (94.7% in erythroid and 96.8% in lymphoid cells), compared to TSSs of tissue-specific genes (77.5% in erythroid and 82.5% in lymphoid cells). CGIs accounted for most of the CFP1 occupancy at TSSs, an observation recapitulated in both gene classes and both cell types, and CFP1 peaks were biased for CGI TSSs in all gene classes, as expected (OR ≥ 5.9, *p* ≤ 1.6 × 10^−12^, Additional file 1: Table S1). Notably, though, housekeeping genes showed increased frequency of CFP1 peaks compared to tissue-specific genes, and this was true in both cell types in both CGI and non-CGI TSSs (Additional file 1: Table S2). Taken together, these findings reinforce the observation that CFP1 occupancy marks active CpG-rich TSSs of both housekeeping and tissue-specific genes, but also reveal that CFP1 is preferentially associated with TSSs of broadly expressed genes whether or not they meet strict criteria for housekeeping genes (see “Methods”).

### CFP1 binds to non-CGI regions associated with transcription

Remarkably, although we observed significant CFP1 bias for CGI TSSs, our data also revealed that CFP1 was bound to a proportion of accessible non-CGI TSSs: for example, 36/78 (46%) and 7/44 (16%) tissue-specific TSSs in ERY and EBV, respectively (Fig. 2d). More than half of non-CGI housekeeping TSSs were occupied by CFP1, consisting of 214/335 (64%) TSSs in ERY and 84/139 (60%) TSSs in EBV (Fig. 2d). In addition to expressed non-CGI TSSs, we found the vast majority of annotated, expressed CGI TSSs occupied by CFP1, including 233/269 (86%) tissue-specific and 5600/5948 (94%) housekeeping TSSs in ERY. Low CpG density, however, was not necessarily correlated with a lack of CFP1 binding, as exemplified by the well-characterised β-globin gene HBB. The HBB locus is CpG-poor (Additional file 1: Fig. S1D), but shows similar CFP1 ChIP signal intensity in erythroid cells to that observed at α-globin gene promoters (Fig. 2e). Experiments using the previously described CFP1 antibody from Abcam confirmed these results, albeit with weaker ChIP signal (Additional file 1: Fig. S5). These findings call into question the previously reported CGI specificity for this protein [14] and raise the possibility that the recruitment of CFP1 at non-CGI sites may occur by protein–protein interactions with pre-existing H3K4me3, due to the reader properties of the plant homeodomain (PHD) of CFP1 [17, 26, 33].

### Coincident CFP1 and Pol II binding at accessible, expressed TSSs

**Fig. 3:**
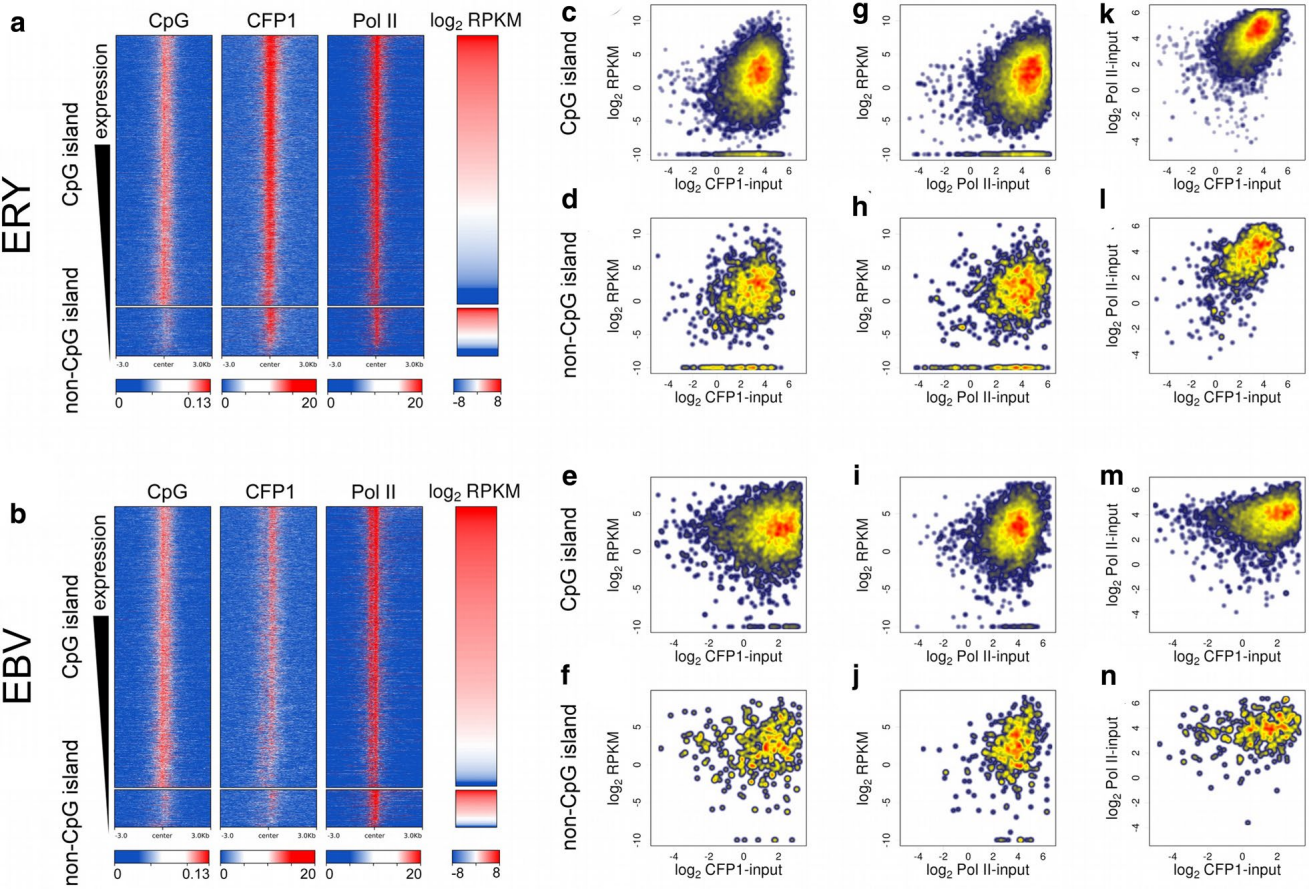
CFP1 binding in TSSs is predictive of expression from accessible TSSs. Upper panels **(a**, **c**, **d**, **g**, **h**, **k**, **l)** represent data in Erythroblasts (ERY) and lower panels **(b**, **e**, **f**, **i**, **j**, **m**, **n)** represent EBV-lymphoblasts (EBV). **a**, **b** Heatmap analysis of CFP1 binding. TSSs are divided into CGI and non-CGI subsets, and then ordered by expression RPKM values. CFP1 and Pol II are measured as read depth normalised to 1x genome-wide, and input-subtracted. **c**–**f** Gene expression level plotted against normalised CFP1 signal (thresholded and log-transformed). Gene expression measure is log_2_ RPKM of all transcripts of the gene combined. **c**, **e** CGI TSSs and **d**, **f** non-CGI TSSs. **g–j** Gene expression level plotted against normalised Pol II signal. **g**, **i** CGI TSSs and **h**, **j** non-CGI TSSs. **k**–**n** Pol II signal plotted against CFP1 signal: **k**, **m** CGI TSSs and **l**, **n** non-CGI TSSs

We next examined more closely the relationship between CFP1, Pol II and expression level using public RNA-seq data sets. We limited our analysis to accessible TSSs (defined by overlap with a 1x-normalised, input-subtracted ATAC-seq signal > 10); this accessibility makes these strong candidate TSSs for the observed expression (Fig. 3a, b). Accessible TSSs of expressed genes in both cell types were occupied with CFP1, regardless of the presence of a CGI (Fig. 3c–f). A few accessible TSSs appeared non-expressed in the available public erythroid data set (GSE74246) while displaying some CFP1 binding in our independent data set (bottom of Fig. 3c, d). We considered it most likely that these are small artefacts due to variability between RNA-seq data sets. Putatively accessible, expressed TSSs were almost universally marked by Pol II (Fig. 3g–j); however, strength of Pol II ChIP signals was only poorly correlated with expression, in line with the observations of others [34]. Plotting Pol II versus CFP1 occupancy at accessible TSSs (Fig. 3k–n), we found that TSSs with increased Pol II intensity also exhibited generally increased CFP1 intensity. The correlation between Pol II and CFP1 intensity at these loci was weak in EBV cells (Pearson *R* = 0.28, *p* < 10^−200^, and *R* = 0.26, *p* = 1.4 × 10^−12^, in CGI and non-CGI TSSs, respectively); however, this correlation was visually identifiable in ERY (Pearson *R* = 0.61, *p* < 10^−200^, and *R* = 0.71, *p* < 10^−200^, in CGI and non-CGI TSSs, respectively). Within the limits of public data sets, these results demonstrate a weak positive relationship between Pol II and CFP1 at accessible, transcribed TSSs.

### Occupancy of CFP1 at enhancers

Unmethylated CpG dinucleotides at TSSs are known to recruit CFP1 through its ZF-CxxC domain [14]. However, recent studies have demonstrated that, in addition, CFP1 can bind all three H3K4 methylation states with varying affinity via its PHD zinc finger domain [17, 26, 33]. Frequent localisation of H3K4me1 at enhancers could therefore predict the presence of CFP1 at enhancers.

**Fig. 4:**
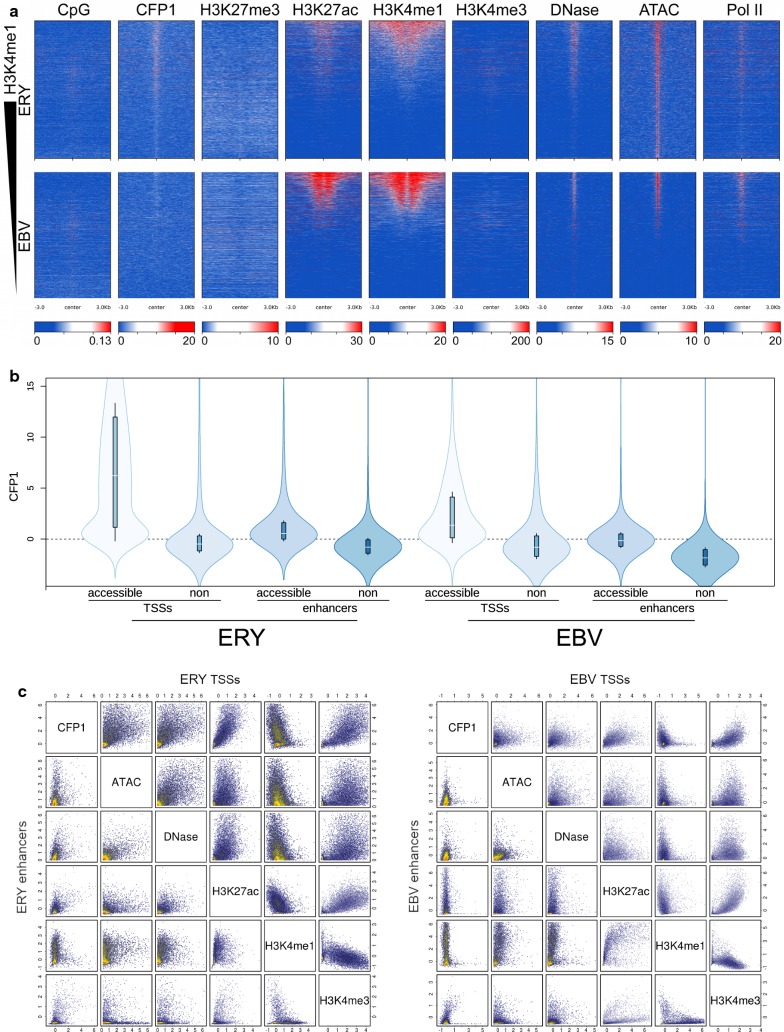
CFP1 binding and histone methylation marks in putative enhancers and comparison to TSSs. **a** Loci sorted by H3K4me1 signal in 6-kb regions surrounding putative enhancers. Upper panels, ERY; lower, EBV. Note that all data sets, other than CFP1, differ by source between ERY and EBV, which explains prominent differences in signal intensities, for example, of H3K27ac and H3K4me1. **b** Quantitative comparison of signal intensity in TSSs and putative enhancers, above and below median accessibility (as determined by ATAC-seq data), with cell types indicated. **c** Plots of ChIP signals for erythroid (left) and lymphoid cells (right), in putative enhancers (lower left graphs) and TSSs (upper right graphs)

To address localisation of CFP1 at enhancers, putative enhancers were defined by open chromatin (ATAC-seq) and the presence of Pol II in non-TSS regions (Fig. 4a, see “Methods”). Elevated DNase hypersensitivity, strong bimodal H3K4me1 signal and H3K27ac surrounding these loci confirmed that these sites are likely active regulatory regions. The presence of little or no H3K4me3 and separation from known TSSs rules out a role for these sites as alternative promoters. The elevated ATAC-seq signal in these regions was largely tissue specific, with 10,548 and 3956 putative enhancers identified in erythroid and lymphoid cells, respectively; only 480 overlaps were observed between these sets of putative enhancers. It should be noted that these putative enhancers represent only a stringent subset with an arbitrarily high ATAC-seq signal, and thus they represent the best-supported candidate enhancers based on the data sets available.

Elevated CFP1 signal was observed at putative enhancers in both cell types, and heatmap evidence suggested qualitative association between CFP1 and signatures of open chromatin (elevated ATAC-seq, DNase-seq, H3K27ac and H3K4me1 signals, Fig. 4a). Indeed, averaged in 2-kb windows around putative enhancers, putative enhancers with above-median ATAC-seq and DNase-seq signals showed elevated CFP1 signal (*p* < 10^−200^, *t* test, both cell types); this, however, was less intense than in accessible TSSs (Fig. 4b). Furthermore, the correlation of CFP1 signal with chromatin accessibility signals was poorer in enhancers than in TSSs for both cell types (Fig. 4a, c). Nonetheless, CFP1 signal intensity was high at putative enhancers associated with a CpG island. Furthermore, intergenic CFP1 peaks (Fig. 1b) showed strong, non-random overlap with our set of putative enhancers. In erythroid cells, 444/1673 CFP1 peaks were colocalised with putative enhancers, which cover 0.16% of the genome (166-fold overrepresented, *p* < 10^−200^, binomial test); in lymphoid cells a weaker but specific overrepresentation was noted, with 159/5488 intragenic CFP1 peaks colocalising with this stringently defined set of putative enhancers (18.1-fold overrepresented, *p* = 1.5 × 10^−137^). These findings demonstrate the presence of CFP1 at enhancers and demonstrate that its association with enhancers is specific.

### CFP1 colocalises with members of the SET1 complexes

**Fig. 5:**
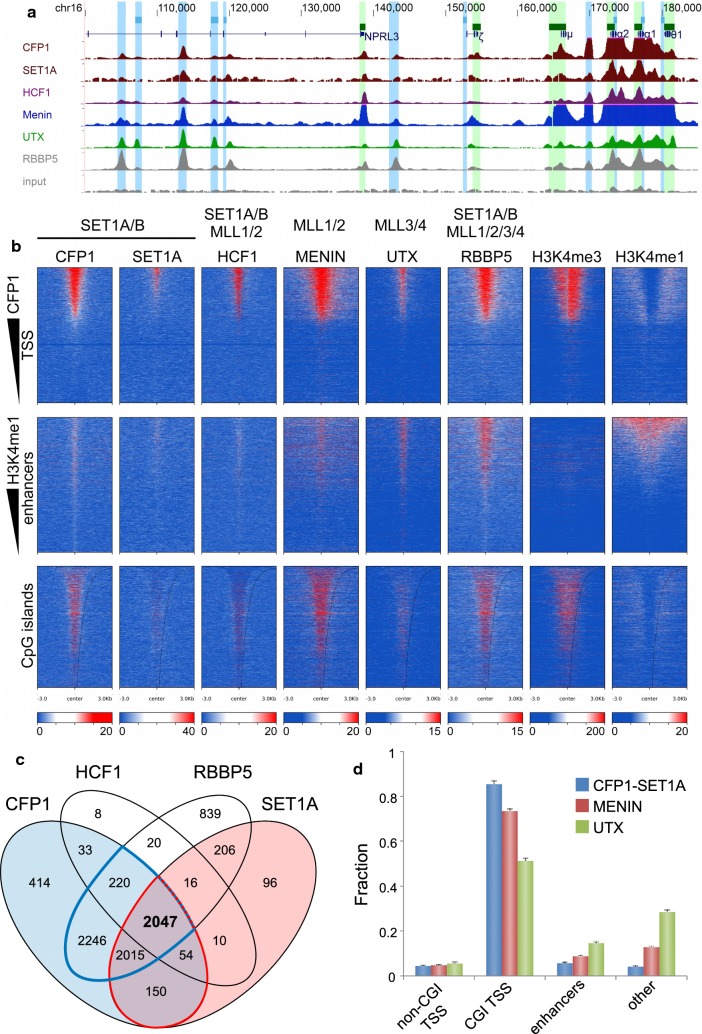
Distribution of TrxG components in erythroid cells. **a** The α-globin locus, with shaded areas indicating CGIs (green) and putative regulatory regions (blue). ChIP signal names indicated to left. Pileups are shown scaled to 1x genome coverage, with full scale 0–50 × depth. **b** Heatmaps showing normalised/input-subtracted erythroid signals for CFP1 and other TrxG subunits. ChIP indicated directly above each heatmap, and TrxG complex indicated above that. Upper, TSSs; middle, putative enhancers; and lower, CGIs (ordered by CGI length). **c** Colocalisation of subunits of SET1A methyltransferase complexes in a high-confidence peak-set. Light blue and light red regions represent regions with CFP1 and SET1A peaks, respectively. Red outline represents colocalisation between CFP1 and SET1A. Blue outline represents CFP1 peaks colocalised with RBBP5. Number in bold represents overlap of all four SET1A complex subunits (CFP1, SET1A, HCF1, and RBBP5). **d** Specialisation of TrxG complexes for enhancers and TSSs. “Other” regions are regions containing peaks of at least two TrxG complex subunits that colocalised neither to TSSs nor putative enhancers defined earlier in the study. SET1A/B complexes are represented by CFP1/SET1A, MLL1/2 complexes by MENIN and MLL3/4 by UTX. Note: error bars are derived by assuming that similarly prepared ChIP experiments give a number of peaks governed by Poisson statistics

The chromatin-binding properties of CFP1, aided by a SET1 subunit, anchor SET1A/B complexes in accessible chromatin [12, 13, 17, 26, 35], and these SET1A/B—along with other TrxG—complexes are responsible for H3K4 methylation in accessible chromatin [13, 18–20, 27, 28, 36–38]. To investigate this, we performed additional ChIP-seq experiments in erythroid cells. We examined genomic occupancies of SET1A (SETD1A) and Host Cell Factor 1 (HCF1/HCFC1), subunits of the SET1A complexes (Fig. 5, Additional file 1: Fig. S6), and RBBP5, a core subunit of all TrxG complexes [11, 39].

To probe the composition of these complexes, we first asked to what extent observed peaks of CFP1, SET1A, HCF1 and RBBP5 were consistent with known composition of SET1A complexes. Upon examination of these ChIP peak overlaps within a high-confidence subset of peak regions (Fig. 5c, see “Methods”), three observations supported the concept that these proteins are subunits of the same complex. First, we observed strong colocalisation between SET1A and CFP1 such that 92.9% (4266/4594) of SET1A peaks were colocalised with 59.4% of the 7179 CFP1 peaks (red outline, Fig. 5c). This observation is consistent with a role for CFP1 as a subunit recruiting SET1A and other proteins to bind to DNA. Furthermore it is consistent with the known difference between SET1A and SET1B localisation [38]: excess CFP1 peaks likely are accounted for by SET1B complexes. Secondly, we noted that 90.9% (6528/7179) of CFP1 peaks were colocalised with RBBP5 (blue outline, Fig. 5c), which is known to form part of a catalytic ASH2L-RBBP5 heterodimer that activates methyltransferase activity in TrxG complexes [36]. This observation suggests a near-complete association of genome-bound CFP1 with RBBP5. Thirdly, we found that a large majority (85.0%, 2047/2408) of HCF1 peaks were colocalised with CFP1, SET1A and RBBP5 (bold font, Fig. 5c). This and the fact that HCF1 colocalisation accounted for a substantial fraction of these three other peak-sets is consistent with CFP1, HCF1 and RBBP5 being obligate members of SET1A complexes. Taken together, this analysis shows that in regions of high-confidence ChIP signals: (1) there is intimate and specific association between SET1A complex components; (2) genomic SET1A and HCF1 are largely or entirely colocalised with CFP1; and (3) the vast majority of genome-bound CFP1 is associated with RBBP5.

### CFP1-SET1A complexes are specialised to TSSs relative to other TrxG complexes

Notably, the above analysis of SET1A complex subunits showed that RBBP5 had a small but substantial fraction of remaining peaks not colocalised with these subunits, suggestive of additional genomic roles for this protein. Therefore, in an expanded analysis, we considered colocalised SET1A and CFP1 (CFP1-SET1A) as representative of SET1A complexes, Menin as a representative of MLL1/2 complexes and UTX as a representative of MLL3/4 complexes (Additional file 1: Fig. S7). This analysis demonstrated that the excess of RBBP5 peak regions unaccounted for by CFP1-SET1A was accounted for by representatives of the MLL1/2 and MLL3/4 complexes. It also showed that genomic occupancy of HCF1 was almost completely accounted for by overlaps with CFP1-SET1A and Menin, which are representatives of SET1 and MLL1/2, respectively. These observations support the view that CFP1, SET1A, HCF1, Menin, UTX and RBBP5 represent a set of proteins whose subunit interactions define compositionally distinct complexes that nevertheless overlap in their genomic occupancy.

We next asked if genomic occupancy of CFP1-SET1A complexes was specialised relative to other TrxG complexes. Specifically, we hypothesised that CFP1-SET1A complexes could differ from other TrxG complexes in their localisation to genomic TSSs and putative enhancers defined earlier. Removing from consideration the small numbers of exclusive CFP1-SET1A (*n* = 9), Menin (*n* = 583) and UTX (*n* = 507) peak regions not colocalising with other TrxG complex subunits (Additional file 1: Fig. S7), we found evidence for substantial site specialisation by the various TrxG complexes (Fig. 5d). CFP-SET1A, Menin and UTX peaks were allocated similarly (approximately 5%) to non-CGI TSSs. In CGI TSSs, all were present at high but differing proportions: 86% of CFP1-SET1A peaks, representing SET1A complexes, were allocated to CGI TSSs, compared to 74% of Menin peaks, representing MLL1/2 complexes, and 51% of UTX peaks, representing MLL3/4 complexes. In our previously defined enhancers, we found the opposite, with putative enhancers representing 6% of CFP1-SET1A peaks, 9% of Menin (MLL1/2) peaks and 15% of UTX (MLL3/4) peaks. In other non-TSS sites of colocalised ChIP peaks, where our conservative criteria did not detect active chromatin, the pattern at enhancers was recapitulated, suggesting that many of these sites act as enhancers. Interestingly, 1409 loci (Additional file 1: Fig. S7) that were external to CFP1-SET1A peaks nevertheless harboured UTX, RBBP5 and Menin (a member of MLL1/2 complexes). Approximately half of these (*n* = 672) were at TSSs. MLL2 is known to deposit H3K4me3 to establish bivalent domains [27, 28, 40], and UTX is a demethylase known to remove repressive H3K27me3 marks [41]. Finding these proteins colocalised is consistent with physical interaction of UTX with MLL2 complex, a phenomenon postulated from co-immunoprecipitation experiments in differentiated cell lines [40]. Taken together, this analysis of regions of high-confidence ChIP signals shows: (1) TrxG subunits are bound to the genome mostly as multi-protein complexes, and it is mostly the presence of these complexes that gives rise to the observed ChIP signals; (2) although components of all TrxG complexes may be found at TSSs, CFP1-SET1A complexes are specialised to TSSs relative to other TrxG complexes; and (3) TrxG complex subunits whose functions include establishment of H3K4me3 at bivalent domains and clearing of the repressive H3K27me3 mark are recruited to sites that do not include CFP1.

### CFP1 occupancy is associated with specific (epi)genomic features and sequence motifs

Given the specificity with which CFP1/SET1A complexes bind in the genome, we next asked what sequence-level determinants might account for this. Therefore, CFP1 peaks were analysed for motif enrichment using homer [42], and ten optimised motifs, five from CGI-associated CFP1 peaks and five from non-CGI peaks, were identified (Additional file 1: Table S3, see “Methods”). Four of five motifs derived from non-CGI peaks lacked a CpG dinucleotide, and four of these five motifs were also strongly biased or exclusive for either cell type, whereas the fifth was of low complexity (Additional file 1: Table S3); these results demonstrate that CFP1 is binding in a cell type-specific manner in non-CGI regions. On the other hand, all five motifs optimised in CGI-associated CFP1 peaks contained a CpG dinucleotide and were found at high frequency in CFP1 peak-sets in both EBV and ERY cell types (Additional file 1: Table S3). Genome-wide however, an average of only 4% of these five CGI-associated motifs (ranging from 2.9 to 6.4%) fell within CGIs, similar to the genomic fraction of CpGs (7.0%) falling within CGIs. This result emphasises the known importance of the CpG dinucleotide, which is bound by CFP1 through its ZF-CxxC domain [17].

To develop a global model of the features specifying CFP1 genomic occupancy, we then analysed the correlation of CFP1 ChIP signal with a number of genomic signals in addition to genomic CpG density in erythroid cells. We considered: (1) that CFP1 is also known to bind all methylation states of H3K4 through its PHD finger domain [26, 33]; (2) that SET1 proteins have been shown to enhance CFP1 interactions with DNA [17]; and (3) that HCF1 and RBBP5 are additional subunits of this complex [12, 36]. These ChIP intensities and CpG density were averaged in 2-kb windows centred on TSSs and putative enhancers (Figs. 1a, 4 and 5; *n* = 41,167 non-redundant loci). To test the sufficiency of these features to explain CFP1 binding, we computed the correlation of each feature with CFP1 individually (Additional file 1: Table S4). Across all 41,167 TSSs and enhancers (irrespective of chromatin state), all features except H3K4me1 were correlated with the CFP1 signal (Pearson *R*^2^ > 0.1, Additional file 1: Table S4). However, restricting our calculations to TSSs and enhancers having top 10% chromatin accessibility scores (4117 sites, Additional file 1: Fig. S8, see “Methods”), only three features were still highly correlated with the CFP1 signal: H3K4me3, SET1A and CpG density. Individually, these features could explain 28.6, 16.0 and 15.2% of the CFP1 signal, respectively, but incorporated in a linear regression model, these features accounted for 47.7% of CFP1 signal (Additional file 1: Table S5, Additional file 1: Fig. S9). This result shows that these three features contribute mostly additively to CFP1 binding in accessible chromatin regions.

## Discussion

Previous studies have demonstrated a key role for CFP1 in the differentiation of mouse ES cells [22], murine early development [21] and haematopoiesis in mouse and fish [23–25]. Ablation of CFP1 reduces H3K4me3 at CGIs, especially downstream of the TSS, with only moderate changes in gene expression [15, 17]. Nevertheless, CFP1 lacking DNA-binding properties can target regions with pre-existing low levels of H3K4me3 [15]. Interaction of CFP1 with H3K4me3 occurs via its PHD domain [17, 26]. Here we have studied the genome-wide occupancy of CFP1 in two human haematopoietic lineages. In agreement with a recent genome-wide study in mouse ES cells [17], we found CFP1 associated with transcription from CGI promoters in human haematopoietic lineages, with CFP1 prominently occupying TSSs of both housekeeping and tissue-specific genes.

Several features have been found to be correlated with CFP1 binding to DNA or chromatin. Some of the earlier analyses pointed to the ZF-CXXC domain and its role in binding to CpG dinucleotides [14, 16, 43]. A more recent study has shown a role for SET1A and methylated H3K4 in addition to unmethylated CpGs [17]. Our analysis of these and other (epi)genomic features found that CpG density, H3K4me3 intensity and SET1A intensity were the most prominently correlated with CFP1 occupancy and are sufficient to explain approximately 48% of CFP1 binding in open chromatin. This result is particularly compelling in view of expected variability in ChIP-seq signals and implies that CFP1 genomic occupancy is largely determined by: (1) binding of its ZF-CxxC domain to CpG dinucleotides; (2) interaction of its PHD finger domain with methylated forms of H3K4; and (3) stabilisation of binding by interaction with SET1. This multifaceted model of CFP1 binding may explain the association of CFP1 with expression in both the α- and β-globin TSSs, which differ strongly in CpG density. In agreement with the observations of others [35], this model can explain the tight association we found between ChIP peaks of SET1A/B complex members in high-confidence regions of strong CFP1, SET1A, HCF1, Menin, UTX or RBBP5 ChIP peaks.

Focusing on enhancers, we found specific occupancy of CFP1 at enhancers in both cell types together with positive correlation with chromatin accessibility. Moreover, enrichment of SET1A and its strong correlation with CFP1, including at enhancers, support the stabilisation of CFP1 at enhancers. SET1A is known to interact with CFP1 and immobilise CFP1 to DNA [17]. Weaker correlation between CFP1 and chromatin accessibility in enhancers compared to TSSs could be explained by reduced affinity of CFP1 for H3K4me1 compared with H3K4me3 [17, 33] and depletion of CGIs in most enhancers.

In addition to the understanding of the mechanisms by which CFP1-containing complexes bind to open chromatin in steady state, an enduring question is how transcription is dynamically regulated in a cell type-specific manner. Early genome-wide studies showed monovalent H3K27me3 is a mark of PcG binding at TSSs and is associated with silencing, whereas monovalent H3K4me3 at TSSs is associated with high expression in mouse ES cells. However, TSSs of genes poised for expression are found in an intermediate bivalent (H3K27me3/H3K4me3) state, which arises as a result of targeted H3K4 methyltransferase activity [44–49]. Mutual exclusivity of repressive H3K27me3 and CFP1 genome-wide, as reported here, thus rules out CFP1-SET1 as the methyltransferase complex responsible for the transition of TSSs from repressed to bivalent. Instead, recent evidence shows that MLL2 methyltransferase activity is required for initial trimethylation establishing bivalent promoters in ES cells, whereas MLL1 is at least in part redundant [27, 28].

In differentiated cells, CFP1-SET1 complexes have been found by others to be responsible for most of the H3K4me3 deposition at CGI [16]. In that light, it is interesting that we observed colocalisation between CFP1-SET1A and Menin (a component of MLL2 complex). Perhaps even more enlightening is our observation of substantial colocalisation between Menin, RBBP5 (a component of all TrxG complexes) and UTX, suggesting the possibility of frequent co-recruitment of MLL2 and UTX at promoters and enhancers lacking CFP1-SET1A. Recent evidence also suggests the existence of a complex involving MLL2 and UTX [40]. UTX is an H3K27me3 demethylase at target genes [41, 50, 51] and at poised enhancers as part of MLL3/4 complexes [35, 40, 52] and also as a chromatin-remodelling factor [53]. Accordingly, our results suggest that before formation of CFP1-SET1 complexes at TSSs, UTX is recruited together with MLL2 to remove repressive H3K27me3 and deposit initial H3K4me3 [27, 28]. This initial deposition of H3K4me3 by MLL2 complexes could provide an anchor for subsequent binding of CFP1-SET1 complexes, which may colocalise with MLL2 to maintain high levels of H3K4me3, irrespective of CpG content. This model presents a coherent explanation for the transition from repressed to open chromatin in keeping with our observed mutual exclusivity of the H3K27me3 mark and CFP1 binding. This model also places into context the evidence that in differentiated cells methyltransferase activity in bivalent domains switches from MLL2 to SET1A [54] and thus helps to broaden our view of key elements involved in gene regulation through chromatin modification. Further analyses of mutations in key proteins in this regulatory network will be necessary to expand on mechanistic insights into CFP1 functions. These might reveal CFP1 trafficking, protein–protein interactions and their timing, and molecular stoichiometry involved in CFP1-dependent gene regulation. Such follow-up studies might also address the relationship between genomic CFP1 distribution, genome-wide transcriptional states and RNA abundance.

## Conclusions

In view of its known specificity for binding to unmethylated CpG dinucleotides, this study revealed an unexpected association of CFP1 with expressed TSSs, even in the absence of CGIs. Furthermore, CFP1 was associated with Pol II binding at expressed TSSs, regardless of CGI status, and this was observed in both cell types. Intriguingly, in addition to strong occupancy at TSSs, CFP1-SET1A complexes were also found at other accessible chromatin regions, including putative enhancers, which were highly enriched in weaker CFP1 peaks. Colocalisation of subunits of other TrxG complexes with CFP1-SET1A complexes was also observed in both TSSs and enhancers, though with differential preference, suggesting partial redundancy of TrxG complexes in agreement with the observations of others in pluripotent stem cells [27].

## Methods

### Primary cells

Primary erythroid cells and newly generated EBV-infected B-lymphoblasts were obtained as previously described [55]. Public EBV ChIP data sets are from the human EBV-infected B lymphocyte cell line GM12878.

### ChIP assay

ChIP was performed as previously described [56]. For CFP1, HCF1, RbBP5, hSET1 (SETD1A), Menin and UTX, chromatin was first cross-linked with ethylene glycol bis(succinimidyl succinate) (EGS) [29] in PBS at a final concentration of 2 mM for 60 min at RT. Formaldehyde (CH_2_O) was then added at a final concentration of 1% for 15 min at RT and samples were sonicated over 20 min (10 × 30-second episodes) at 4 °C to cleave genomic DNA (Bioruptor, Diagenode). Input data sets were matched with ChIP-seq samples. Antibodies used are: H3K27me3 (07-449) (Millipore); HCF1 (A301-400A), RbBP5 (A300-109A), hSET1 (SETD1A, A300-289A), Menin (A300-105A), UTX (A302-374A) from Bethyl Labs and CFP1 (CGBP, ab56035) from Abcam. The non-commercial CFP1 antibody was kindly provided by Prof. Robert Roeder. Real-Time PCR was performed using primers and probes (5’FAM-3’TAMRA) for the murine and human α-globin locus described previously [57, 58]. Each ChIP was performed as two independent experiments and quality was assessed by qPCR. Libraries and sequencing (ChIP-seq) were performed using the standard Illumina kits and protocols.

### DNaseI assay and DNaseI sequencing

Nuclei from human primary erythroid cells were digested with increasing concentrations of DNaseI (Roche) as previously described [59]. DNA (1.5 µg) from the mid-phase digestions was blunt-ended with T4 DNA Polymerase (NEB) and prepared for Illumina HiSeq 2500 sequencing.

### Data sources, protocols and analysis

Sources of ChIP-seq data are shown in Table 1.

**Table 1:** Experiments, protocols, read counts and GEO accession numbers

Cell type	Data set	Crosslinking/Reference	Read count (paired)	GEO accession
Cell type	Data set	Crosslinking/Reference	Read count (paired)	GEO accession
Erythroblasts	CFP1	EGS + CH_2_O	38,713,222 unpaired	GSE114084
Erythroblasts	CFP1 (Abcam)	EGS + CH_2_O	38,789,474 unpaired	GSE114084
Erythroblasts	H3K27ac	[60]	54,716,349 unpaired	GSE70660
Erythroblasts	H3K27me3	EGS + CH_2_O	163,108,458 paired	GSE114084
Erythroblasts	H3K4me1	[60]	26,492,230 unpaired	GSE70660
Erythroblasts	H3K4me3	[61]	14,926,093 unpaired	GSE36985
Erythroblasts	DNase-seq	N/A	281,771,876 paired	GSE114084
Erythroblasts	ATAC-seq	[62]	228,886,766 paired	GSE74912
Erythroblasts	Pol II	[61]	106,164,823 unpaired	GSE36985
Erythroblasts	HCF1	EGS + CH_2_O	179,476,544 paired	GSE114084
Erythroblasts	MEN1	EGS + CH_2_O	81,145,272 paired	GSE114084
Erythroblasts	RBBP5	EGS + CH_2_O	90,587,516 paired	GSE114084
Erythroblasts	SET1A	EGS + CH_2_O	93,079,696 paired	GSE114084
Erythroblasts	UTX	EGS + CH_2_O	65,366,066 paired	GSE114084
Erythroblasts, default input data set	Input 1	EGS + CH_2_O	24,173,450 paired	GSE114084
Erythroblasts, matched with HCF1, SET1A	Input 2	EGS + CH_2_O	86,858,360 paired	GSE114084
Erythroblasts, matched with MEN1	Input 3	EGS + CH_2_O	109,489,716 paired	GSE114084
EBV-transformed B cells	CFP1	EGS + CH_2_O	39,009,169 unpaired	GSE114084
EBV-transformed B cells	CFP1 (Abcam)	EGS + CH_2_O	41,374,998 unpaired	GSE114084
GM12878	H3K27ac	[63]	463,073,456 paired	GSE50893
GM12878	H3K27me3	[63]	480,207,766 paired	GSE50893
GM12878	H3K4me1	[63]	271,241,104 paired	GSE50893
GM12878	H3K4me3	[63]	268,581,398 paired	GSE50893
GM12878	DNase-seq	[64]	400,610,386 unpaired	GSE32970
GM12878	ATAC-seq	[65]; first 24 M read pairs in SRA data sets SRR3336945-52	192,000,000 paired	GSE79921
GM12878	Pol II	[66]	60,061,473 unpaired	GSE19486
EBV-transformed B cells, default input data set	Input 1	EGS + CH_2_O	19,753,388 paired	GSE114084
GM12878, used with H3K27me3	Input 2	[63]	112,326,958 paired	GSE50893

### ChIP-Seq analysis

Reads were aligned using the hisat2 aligner version 2.0.3 with the --no-spliced-alignment option, but otherwise default parameters. Reads were aligned to a splicing-unaware index of the human GRCh38 genome. ChIP peaks were identified using the ENCODE tool Phantompeakqualtools [67], an R script that called peaks using the SPP library [68] version 1.14. Peaks in each experiment were thresholded by FDR < 0.01, except for H3K27me3 in EBV cells, in which case peaks were thresholded by FDR < 0.1. Narrow peaks were used for analysis of all ChIPs except the H3K27me3 mark, in which case region peaks were analysed. As a confirmation in specific cases, ChIP peaks were secondarily identified using the callpeak function of MACS2, version 2.1.1.20,160,309, with narrow peaks called using a q-value threshold at 0.01, and broad peaks with the --broad-cutoff 0.1 option. Peak colocalisation analysis was carried out using the ChIPpeakAnno package in R, with maximum gap of 1 kb. General genome arithmetic, including peak intersection with TSSs, was carried out using bedtools [69]. ChIP heatmap plots were generated using the bamCompare, computeMatrix and plotHeatmap functions of deepTools version 2.5.4 [70]. Read depths were compared between ChIP-seq and input data sets using bamCompare with flags --scaleFactorsMethod readCount --ratio subtract --binSize 50 --normalizeTo1x 3100000000 --minMappingQuality 30; this normalisation and subtraction was performed on all data sets. To compare CpG content signal with ChIP signals, numbers of CpG sites were counted in 50-bp windows, such that 5 CpG sites was computed as 10% of sites in a 50-bp window. To analyse correlation of two ChIP signals or CpG density and ChIP signals, the given signal distributions were first averaged within 2-kb loci and correlation was computed among loci.

### Identification of a set of putative enhancers

Putative genomic enhancers were identified by a stepwise procedure using Assay for Transposase-Accessible Chromatin (ATAC), Pol II and H3K4me1 signals. First, normalised genome-wide signals for each ChIP were constructed by normalising each pileup to 1x genome coverage, and subtracting similarly normalised input data using the deepTools bamCompare program as described above. Genomic regions > 2 kb from gene TSSs exhibiting ATAC-seq signal value > 10 that also exhibited a Pol II signal value > 5 were accepted as putative enhancers and confirmed by plotting heatmaps of H3K4me1 and H3K27ac around these loci.

### RNA-seq analysis and identification of housekeeping and cell type-specific genes

RNA-seq data from NCBI GEO (accession GSE74246, three erythroblast samples, and GSE88627, four GM12878 EBV-lymphoblast samples) were aligned to a special purpose, splice-aware version of the GRCh38 human genome downloaded from the hisat2 web page (https://ccb.jhu.edu/software/hisat2/index.shtml). The hisat2 aligner, version 2.0.5, was used with default parameters. Analysis of expression was performed in R: read counts for each locus were obtained in unpaired mode using the featureCounts function from the Rsubread package, and reads per kilobase of transcript per million mapped reads (RPKM) values were calculated using the rpkm function from the edgeR package.

Housekeeping genes and candidate tissue-specific genes were identified from processed RNA-seq expression data (16 tissues) warehoused by the EBI Illumina body map resource (NCBI GEO accession GSE30611). Gene RPKM values were downloaded and thresholded at 0.1. A previously reported method [71] developed for identification of housekeeping exons was applied to whole genes to identify a list of housekeeping genes. Briefly, genes with RPKM ≥ 0.1 in all tissues, standard deviation in log_2_(RPKM) < 1, and no tissue with absolute log_2_(RPKM/mean(RPKM)) > 2 (all expression ratios less than 4 in either direction) were termed housekeeping genes. Genes for which either all expression RPKM ≤ 0.1 or for which more than half of the tissues had RPKM values ≤ 10% maximum were considered to be normally non-expressed. The maximal expression RPKM value of these candidate tissue-specific genes was compared to expression RPKM values (averaged across samples) from the analysis of erythroid and lymphoid cell types (above). If a gene was on the list of normally non-expressed genes and its RPKM expression value in erythroid or lymphoid tissue was at least half of this maximum, it was termed tissue-specific in that tissue.

### Analysis of TrxG subunit occupancy in a high-confidence subset of peak regions

To analyse which subunits of TrxG-related methyltransferase complexes colocalised, we compared six ChIP data sets in erythroid cells: CFP1 and SET1A, subunits of SET1A/B complexes; HCF1, a subunit of SET1A/B and MLL1/2 complexes; Menin, a subunit of MLL1/2 complexes; UTX, a subunit of MLL3/4 complexes; and RBBP5, a core subunit of all the complexes. A high-confidence set of peaks in each data set was defined as its most highly enriched tenth percentile, as detected by SPP. Bedtools was used to merge these regions into a non-redundant set of high-confidence peak regions. Subsequently peaks of all strengths detected by SPP were analysed for the presence or absence in these high-confidence regions.

### Analysis of motifs in ChIP-enriched regions

ChIP-enriched regions, identified as peaks by SPP, were analysed for motif enrichment using the homer program [42]. For each ChIP experiment, peaks were divided into two groups by the presence or absence of CGIs. The top 1000 peaks by coverage depth were analysed in each group. Homer selected genomic background sequences matched to target sequences by length and 3-bp oligo content. Homer then used binomial statistics to compare motif frequencies in target and background sequences. It first identified enrichments of 364 known motifs, then identified 75 additional de novo motifs (25 each at sizes of 8, 10 and 12 base pairs). Thus, a total of 300 partially redundant motifs were identified, 150 from CGI-associated CFP1 peaks in both cell types and 150 from non-CGI peaks.

Motifs passing a detection threshold of *p* < 10^−20^ were selected and further optimised by homer, with optimisation carried out separately for CGI and non-CGI peaks. Target sequences used for the optimisation step were combined peaks from ERY and EBV cell types, but with redundant peaks removed. The optimised motifs were clustered by homer and the most significant exemplar in each cluster was reported. The top ten exemplar motifs, five identified from CGI-associated CFP1 peaks and five from non-CGI peaks, were subsequently detected in CGI and non-CGI peaks, respectively. The purpose of this detection was to determine if a given motif, which had been optimised using sequences from both ERY and EBV cells, was biased for ERY or EBV cells; this might occur if the motif reflected genes expressed in that cell type rather than structural binding preference of CFP1.

### Analysis of correlation of ChIP signal intensities with (epi)genomic features

We sought to understand the relationship between the intensity of ChIP and other signals, which might indicate functional interaction. CpG density and erythroid ChIP signals from CFP1, SET1A, HCF1, RBBP5, H3K4me3, H3K4me1, H3K27me3, DNase-seq and ATAC-seq were analysed, and ChIP signals were 1x-normalised and input was subtracted, as described above. Given that normalised and input-subtracted ChIP signals were noisy, each ChIP signal was averaged in a 2-kb region surrounding each TSS and putative enhancer to give a robust representation of the signal strength. The rare case where robust but non-overlapping ChIP-seq peaks are present at the same locus in different ChIP experiments, which would give a spurious apparent colocalisation, was ignored in this analysis. Relative chromatin accessibility was estimated from DNase-seq and ATAC-seq signals. Given that these signals give differing representations of chromatin accessibility, they were combined; first these signals were linearly scaled by the z-score transformation, and then they were averaged. The resulting chromatin accessibility score was therefore more robust and gave equal weight to DNase-seq and ATAC-seq data.

Tests of Pearson’s product moment correlation (*R*^2^) were performed between CFP1 and CpG density, SET1A, HCF1, RBBP5, H3K4me1, H3K4me3 and chromatin accessibility; this was done in all loci and in a subset of loci with upper decile chromatin accessibility scores. Features whose correlation *R*^2^ value was greater than 10% were identified. To test the sufficiency of these features to explain CFP1 occupancy in open chromatin, a linear regression analysis was performed, and this analysis was limited to the top-decile accessible regions.

## Additional file

**Additional file 1.** **Fig. S1**: Analysis of CFP1 binding at individual loci and CpG islands (CGIs). **(A-B)** Analysis of CFP1 binding at the human α-globin locus in expressing and non-expressing cells. **(A)** Real-Time PCR analysis of immunoprecipitated chromatin using CFP1 antibody in human erythroblasts (red) and B-lymphocytes (blue). The y-axis represents enrichment over the input DNA, normalised to a control sequence in the human 18S gene. The x-axis represents the positions of Taqman probes used. The coding sequence is represented by the three exons (Promoter/Ex1, Ex2, Ex3) of the α-globin genes. 218 and hBact denote control sequences adjacent to the CpG islands of the human LUC7L (218) and ACTB promoters. Error bars correspond to ± 1 SD from at least two independent ChIPs. **(B)** Real-Time PCR analysis of immunoprecipitated chromatin using the CFP1 antibody indicated in humanised erythroblasts (normal, +MCS-R2 (left) and mutant, MCS-R2 (right). The y-axis represents enrichment over the input DNA, normalised to a control sequence in the mouse GAPDH gene. CpG Act denotes additional control sequence at the CGI of the mouse ACTB gene. The amplicons highlighted in red represent deleted regions in the humanised mice, for which no PCR signal is observed. Error bars correspond to ± 1 SD from at least two independent ChIPs. **(C)** CFP1 ChIP signal intensity in the top 200 peaks, by antibody and by cell type. Abcam, ab56035 antibody. Roeder, main antibody used in this study. **(D)** Analysis of CGI (green) and non-CGI (blue) transcription start sites (1-kb window, centred on TSS). Gene symbols shown with CpG content of individual loci in parentheses. Greek letters represent individual globin genes. **Fig. S2**: Peak overlaps of CFP1 and marks of active and repressed chromatin in transcription start sites (TSSs). Peaks were detected by MACS2. Venn diagrams show that CFP1 peaks within 1-kb of TSSs are strongly associated with H3K4me3 histone mark and poorly associated with H3K27me3 repressive histone mark. Cell types are **(A)** ERY and **(B)** EBV. Public data sets: * NCBI GEO GSE36985, ** NCBI GEO GSE50893. **Fig. S3**: UCSC tracks showing CFP1 and other ChIP signals in gene loci in erythroblasts (ERY) and EBV-transformed B-lymphoblasts (EBV). Hg38 coordinates for multiple genes, CpG islands (CGI, green boxes), and putative regulatory regions (blue boxes) are shown. CFP1 signals are shown in dark reds, inputs in grey, histone H3 signals in blues and open chromatin marks in greens. All ChIP pileups are scaled to 1x coverage genome-wide and shown in a range 0–50, except CFP1 (Roeder) is shown with extended range and H3K27me3 graphs scaled by 2x. **(A)** Tissue-specific binding of CFP1 to CGI promoters of tissue-specifically expressed genes. Left (chr16), CGI promoters of active genes in alpha globin locus are CFP1-bound in ERY, and unbound in EBV. Flanking regions are included, with known tissue-specific enhancers. Right (chr6), first seven exons of IRF4 locus, active in EBV and inactive in ERY, with CFP1 binding to CGI promoter in EBV only. **(B)** CGI promoters of housekeeping genes are CFP1 bound and unmarked by H3K27me3. Left (chr7), ACTB locus. Right (chr16), LUC7L locus. **(C)** CGI promoter of RHBDF1 locus (chr16) has H3K27me3 mark and the absence of CFP1 binding in both ERY and EBV. **Fig. S4**: Western blot analysis of CGBP (CFP1) expression in mouse and human erythroid and human lymphoid cell types. Whole cell extracts (20 µg) were loaded in each lane (1) mouse ES, (2) U-MEL, (3) I-MEL, (4) mouse primary erythroblasts and (5) human primary T lymphocytes and (6) human primary erythroblasts and separated on a 10% SDS-polyacrylamide gel. CFP1 antibody was used at a 1:1000 dilution. **Fig. S5**: Similar cell type-specific CFP1 read depth at CGI TSS of HBA1 gene and non-CGI TSS of HBB gene. Upper two tracks use the main antibody, and second two tracks use the commercial antibody. Coordinates are from the hg38 human genome build. Read depths are averaged in 50 bp bins and normalised to 1x genome-wide coverage. Blue boxes, known regulatory regions; green box, CGI. **Fig. S6**: Distribution of TrxG components in erythroid cells. Green indicates CGI and blue indicates other putative regulatory regions. All loci transcribed right to left. Pileups are shown scaled to 1x genome coverage, with full scale 0–50x depth. **(A)** Housekeeping genes ACTB, left (chr7), and LUC7L, right (chr16). **(B)** β-globin locus (chr11), **(C)** Non-expressed RHBDF1 locus (chr16). **Fig. S7**: Overlap of TrxG subunit ChIP peaks in a high-confidence subset of regions. SET1A complexes are represented by CFP1-SET1A colocalisation. MLL1/2 complexes are represented by Menin, and MLL3/4 complexes are represented by UTX, respectively. HCF1 is found in SET1A/B and MLL1/2 complexes, and RBBP5 is a member of SET1A/B and MLL1/2/3/4 complexes. Red outline (4220 peaks) shows strong colocalisation of Menin and CFP1-SET1A, accounting for the vast majority (99.5%) of 4242 CFP1-SET1A and half (50.0%) of 8432 Menin peak regions. Majority (87.0%, 2089/2400 peaks) of HCF1 (blue region) is accounted for by approximately half (49.5%, 2089/4220) of regions of Menin-SET1A-CFP1 colocalisation. Regions where either SET1A-CFP1 or Menin or both are colocalised with HCF1 (blue dashed line) accounts for nearly all (99.6%, 2390/2400) HCF1 regions, suggesting that HCF1 bound to DNA is primarily present as part of SET1A/B or MLL1/2 complexes. **Fig. S8**: Chromatin accessibility in TSSs and enhancers in erythroid cells as measured by ATAC-seq and DNase-seq. 1x-normalised, input-subtracted signals from ATAC-seq and DNase were averaged in a 2-kb window about TSSs and putative enhancers. Z-score transformed values for ATAC-seq and DNase-seq at a given locus were averaged. **Fig. S9**: Relationship of CFP1 signal to three predictive factors in top-decile open chromatin regions. A linear combination of CpG density and SET1A and H3K4me3 ChIP signals explains a substantial fraction of variation in CFP1 ChIP signal. **Table S1**: Bias of CFP1 for CGI TSSs in cell types and gene classes. **Table S2**: Bias of CFP1 for housekeeping gene TSSs. **Table S3**: Motifs associated with CFP1 peaks. **Table S4**: Dependence of CFP1 ChIP signal in erythroid cells on covariates putatively associated with its binding. **Table S5**: Analysis of variance of CFP1 signal in top-decile open chromatin regions surrounding TSSs and putative enhancers.
