# Supplementary material for: CpG binding protein (CFP1) occupies open chromatin regions of active genes, including enhancers and non-CpG islands
Source: Epigenetics Chromatin. 2018 Oct 6;11:59. doi: 10.1186/s13072-018-0230-0 (PMC6173865; doi:10.1186/s13072-018-0230-0)
Supplement: Supplementary file 1 — Additional file 1. Fig. S1: Analysis of CFP1 binding at individual loci and CpG islands (CGIs). (A-B) Analysis of CFP1 binding at the human α-globin locus in expressing and non-expressing cells. (A) Real-Time PCR analysis of immunoprecipitated chromatin using CFP1 antibody in human erythroblasts (red) and B-lymphocytes (blue). The y-axis represents enrichment over the input DNA, normalised to a control sequence in the human 18S gene. The x-axis represents the positions of Taqman probes used. The coding sequence is represented by the three exons (Promoter/Ex1, Ex2, Ex3) of the α-globin genes. 218 and hBact denote control sequences adjacent to the CpG islands of the human LUC7L (218) and ACTB promoters. Error bars correspond to ± 1 SD from at least two independent ChIPs. (B) Real-Time PCR analysis of immunoprecipitated chromatin using the CFP1 antibody indicated in humanised erythroblasts (normal, +MCS-R2 (left) and mutant, MCS-R2 (right). The y-axis represents enrichment over the input DNA, normalised to a control sequence in the mouse GAPDH gene. CpG Act denotes additional control sequence at the CGI of the mouse ACTB gene. The amplicons highlighted in red represent deleted regions in the humanised mice, for which no PCR signal is observed. Error bars correspond to ± 1 SD from at least two independent ChIPs. (C) CFP1 ChIP signal intensity in the top 200 peaks, by antibody and by cell type. Abcam, ab56035 antibody. Roeder, main antibody used in this study. (D) Analysis of CGI (green) and non-CGI (blue) transcription start sites (1-kb window, centred on TSS). Gene symbols shown with CpG content of individual loci in parentheses. Greek letters represent individual globin genes. Fig. S2: Peak overlaps of CFP1 and marks of active and repressed chromatin in transcription start sites (TSSs). Peaks were detected by MACS2. Venn diagrams show that CFP1 peaks within 1-kb of TSSs are strongly associated with H3K4me3 histone mark and poorly associated with H3K27me3 repressive hi [file 13072_2018_230_MOESM1_ESM.pdf]

ADDITIONAL FILE 1

CpG binding protein (CFP1) occupies open chromatin regions of active genes, including enhancers and non-CpG islands.

Louie N. van de Lagemaat[1], Maria Flenley[2], Magnus D. Lynch[2,3], David Garrick[4], Simon R. Tomlinson[5], Kamil R. Kranc[5,6] and Douglas Vernimmen[1].

[1] The Roslin Institute, Developmental Biology Division, University of Edinburgh, Easter Bush, Midlothian EH25 9RG, United Kingdom.

[2] MRC Molecular Haematology Unit, Weatherall Institute for Molecular Medicine, University of Oxford, John Radcliffe Hospital, Oxford OX3 9DS, United Kingdom.

[3] Centre for Stem Cells and Regenerative Medicine, King's College London, London WC2R 2LS, United Kingdom.

[4] INSERM, UMRS-1126, Institut Universitaire d'Hématologie, Université Paris, 75010 Paris, France.

[5] MRC Centre for Regenerative Medicine, 5 Little France Drive, University of Edinburgh, Edinburgh EH16 4UU, United Kingdom.

[6] Edinburgh Cancer Research UK Centre, MRC Institute of Genetics and Molecular Medicine, University of Edinburgh, Western General Hospital, Crewe Road South, Edinburgh EH4 2XR, United Kingdom.

A

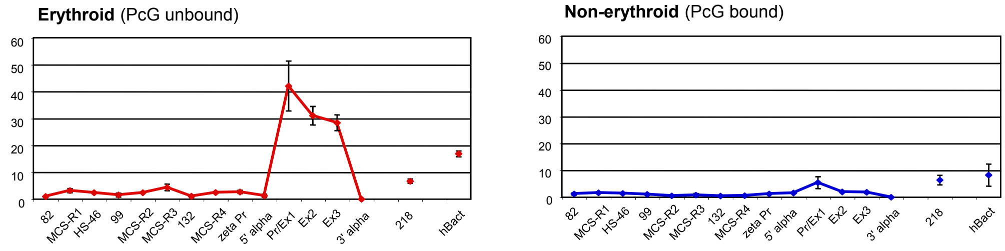
Erythroid (PcG unbound)
Non-erythroid (PcG bound)
60
50
40
30
20
10
0
82
MCS-R1
HS-46
99
MCS-R2
MCS-R3
132
MCS-R4
zeta Pr
5' alpha
Pr/Ex1
Ex2
Ex3
3' alpha
218
hBact

B

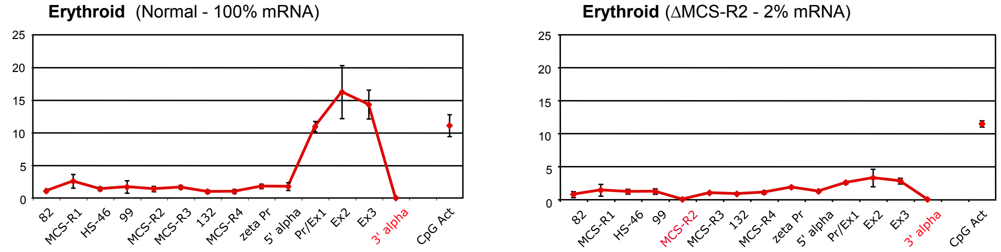
Erythroid (Normal - 100% mRNA)
Erythroid (ΔMCS-R2 - 2% mRNA)
25
20
15
10
5
0
82
MCS-R1
HS-46
99
MCS-R2
MCS-R3
132
MCS-R4
zeta Pr
5' alpha
Pr/Ex1
Ex2
Ex3
3' alpha
CpG Act

C

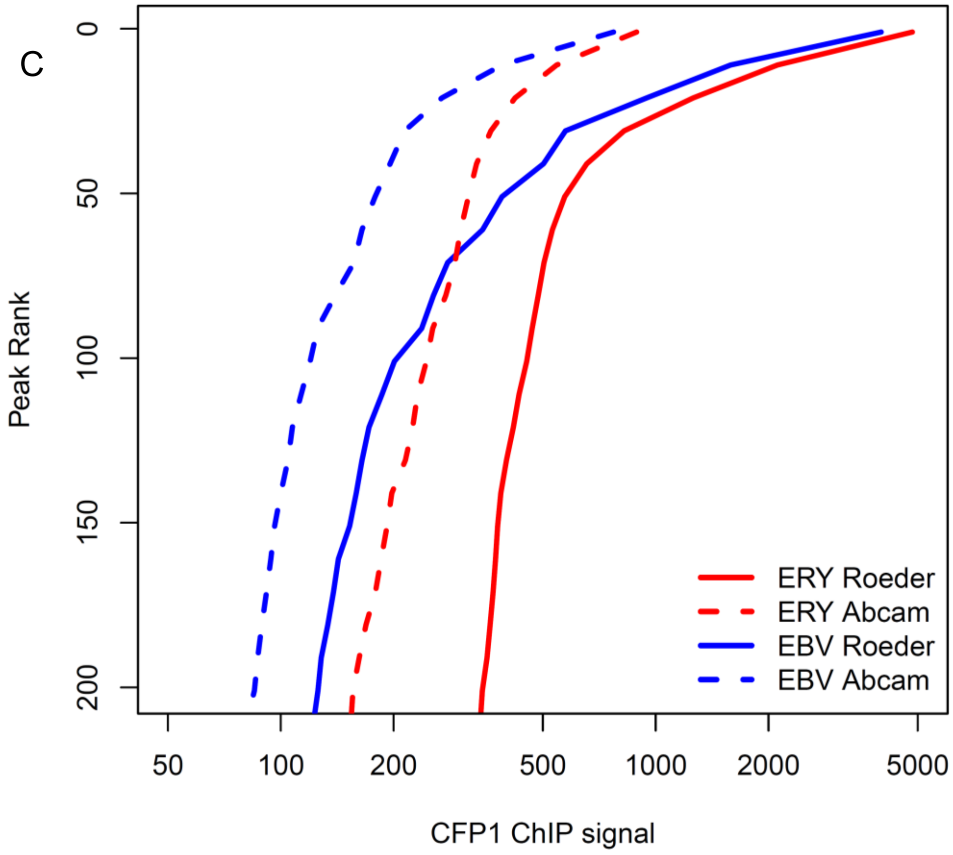
Peak Rank
0
50
100
150
200
50
100
200
500
1000
2000
5000
CFP1 ChIP signal
ERY Roeder
ERY Abcam
EBV Roeder
EBV Abcam

D

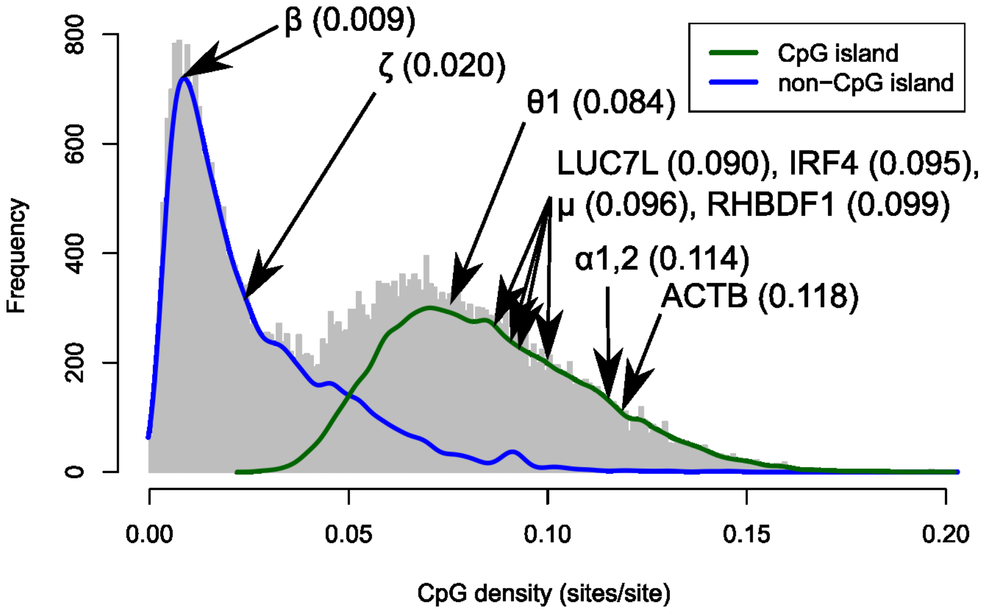

Fig. S1 – Analysis of CFP1 binding at individual loci and CpG islands (CGIs). (A-B) Analysis of CFP1 binding at the human α-globin locus in expressing and non-expressing cells. **(A)** Real-Time PCR analysis of immunoprecipitated chromatin using CFP1 antibody in human erythroblasts (red) and B-lymphocytes (blue). The y axis represents enrichment over the input DNA, normalised to a control sequence in the human 18S gene. The x axis represents the positions of Taqman probes used. The coding sequence is represented by the three exons (Promoter/Ex1, Ex2, Ex3) of the α-globin genes. 218 and hBact denote control sequences adjacent to the CpG islands of the human LUC7L (218) and ACTB promoters. Error bars correspond to ± 1 s.d. from at least two independent ChIPs. **(B)** Real-Time PCR analysis of immunoprecipitated chromatin using the CFP1 antibody indicated in humanized erythroblasts (normal, +MCS-R2 (left) and mutant, MCS-R2 (right). The y axis represents enrichment over the input DNA, normalised to a control sequence in the mouse GAPDH gene. CpG Act denotes additional control sequence at the CGI of the mouse ACTB gene. The amplicons highlighted in red represent deleted regions in the humanised mice, for which no PCR signal is observed. Error bars correspond to ± 1 s.d. from at least two independent ChIPs. **(C)** CFP1 ChIP signal intensity in the top 200 peaks, by antibody and by cell type. Abcam, ab56035 antibody. Roeder, main antibody used in this study. **(D)** Analysis of CGI (green) and non-CGI (blue) transcription start sites (1 kb window, centred on TSS). Gene symbols shown with CpG content of individual loci in parentheses. Greek letters represent individual globin genes.

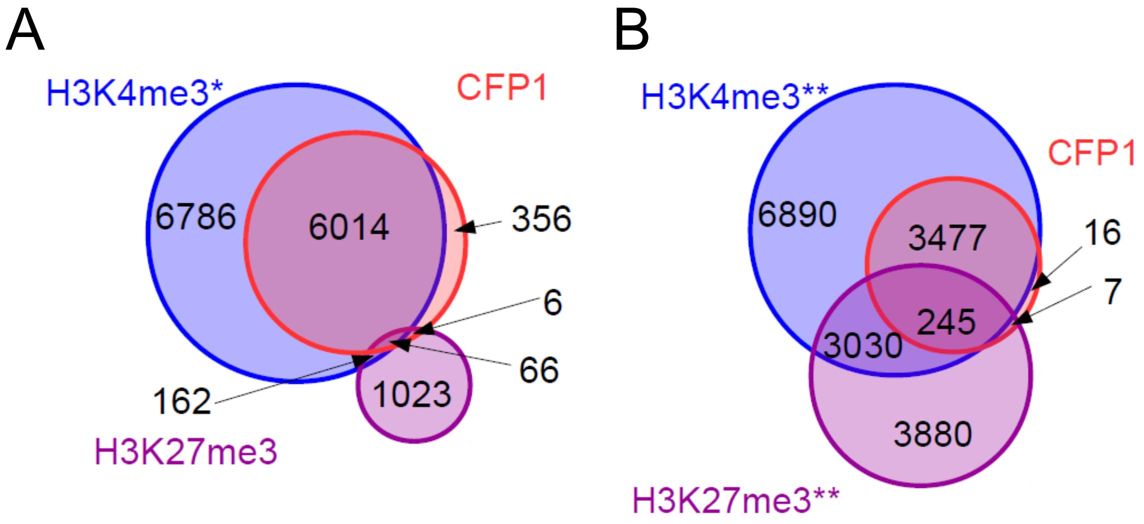

Fig. S2 – Peak overlaps of CFP1 and marks of active and repressed chromatin in transcription start sites (TSSs). Peaks were detected by MACS2. Venn diagrams show that CFP1 peaks within 1-kb of TSSs are strongly associated with H3K4me3 histone mark and poorly associated with H3K27me3 repressive histone mark. Cell types are **(A)** ERY and **(B)** EBV. Public datasets: * NCBI GEO GSE36985, ** NCBI GEO GSE50893.

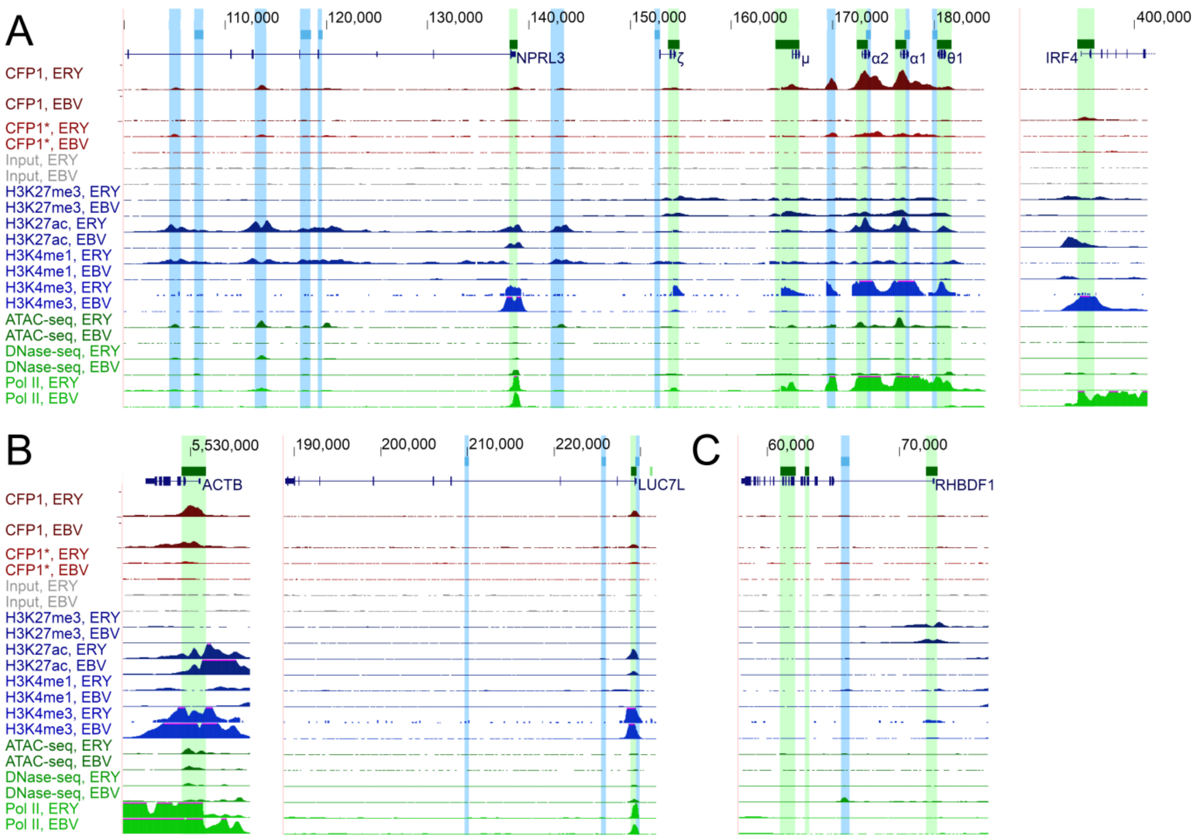

Fig. S3 – UCSC tracks showing CFP1 and other ChIP signals in gene loci in erythroblasts (ERY) and EBV-transformed B lymphoblasts (EBV). Hg38 coordinates for multiple genes, CpG islands (CGI, green boxes), and putative regulatory regions (blue boxes) are shown. CFP1 signals are shown in dark reds, inputs in grey, histone H3 signals in blues, and open chromatin marks in greens. All ChIP pileups are scaled to 1x coverage genome-wide and shown in a range 0-50, except CFP1 (Roeder) is shown with extended range and H3K27me3 graphs scaled by 2x. **(A)** Tissue-specific binding of CFP1 to CGI promoters of tissue-specifically expressed genes. Left (chr16), CGI promoters of active genes in alpha globin locus are CFP1-bound in ERY, and unbound in EBV. Flanking regions are included, with known tissue specific enhancers. Right (chr6), first seven exons of IRF4 locus, active in EBV and inactive in ERY, with CFP1 binding to CGI promoter in EBV only. **(B)** CGI promoters of housekeeping genes are CFP1 bound and unmarked by H3K27me3. Left (chr7), ACTB locus. Right (chr16), LUC7L locus. **(C)** CGI promoter of RHBDF1 locus (chr16) has H3K27me3 mark and absence of CFP1 binding in both ERY and EBV.

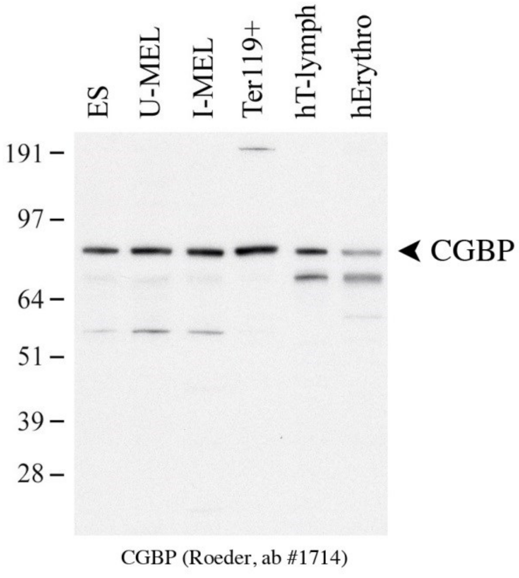

Fig. S4 – Western blot analysis of CFP1 (CGBP) expression in mouse and human erythroid and human lymphoid cell types. Whole cell extracts (20 µg) were loaded in each lane (1) mouse ES, (2) U-MEL, (3) I-MEL, (4) mouse primary erythroblasts and (5) human primary T lymphocytes and (6) human primary erythroblasts and separated on a 10% SDS-polyacrylamide gel. CFP1 antibody was used at a 1:1000 dilution.

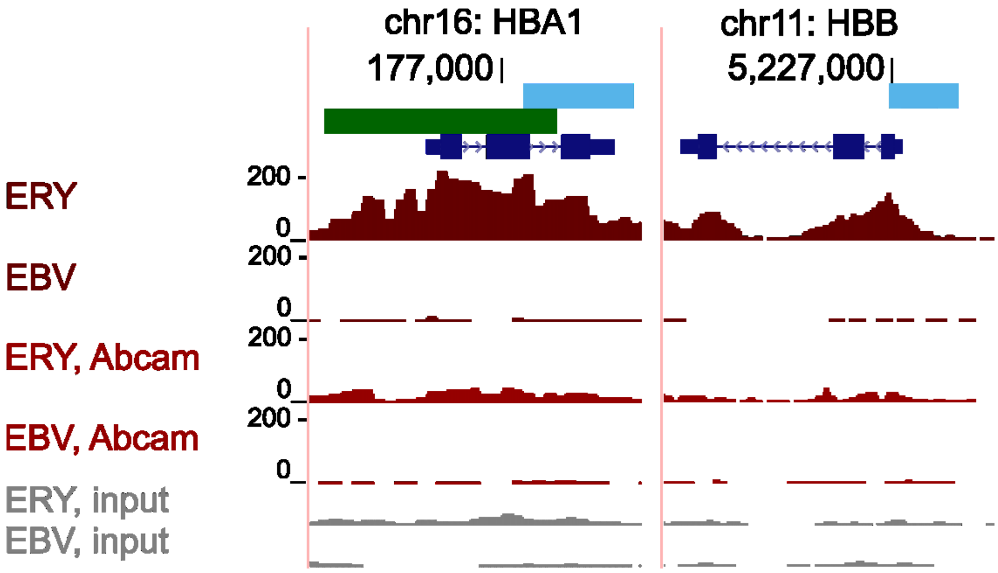

Fig. S5 – Similar cell type-specific CFP1 read depth at CGI TSS of HBA1 gene and non-CGI TSS of HBB gene. Upper two tracks use the main antibody, and second two tracks use the commercial antibody. Coordinates are from the hg38 human genome build. Read depths are averaged in 50bp bins and normalised to 1x genome-wide coverage. Blue boxes, known regulatory regions; green box, CGI.

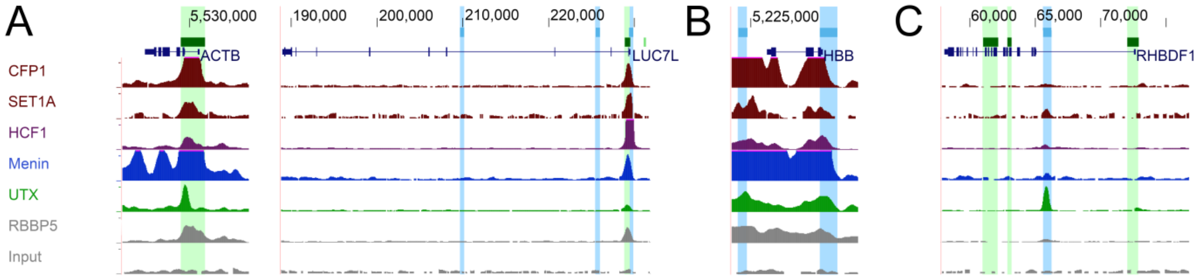

Fig. S6 – Distribution of TrxG components in erythoid cells. Green indicates CGI and blue indicates other putative regulatory regions. All loci transcribed right to left. Pileups are shown scaled to 1x genome coverage, with full scale 0-50x depth. **(A)** Housekeeping genes ACTB, left (chr7), and LUC7L, right (chr16). **(B)** β-globin locus (chr11), **(C)** Non-expressed RHBDF1 locus (chr16).

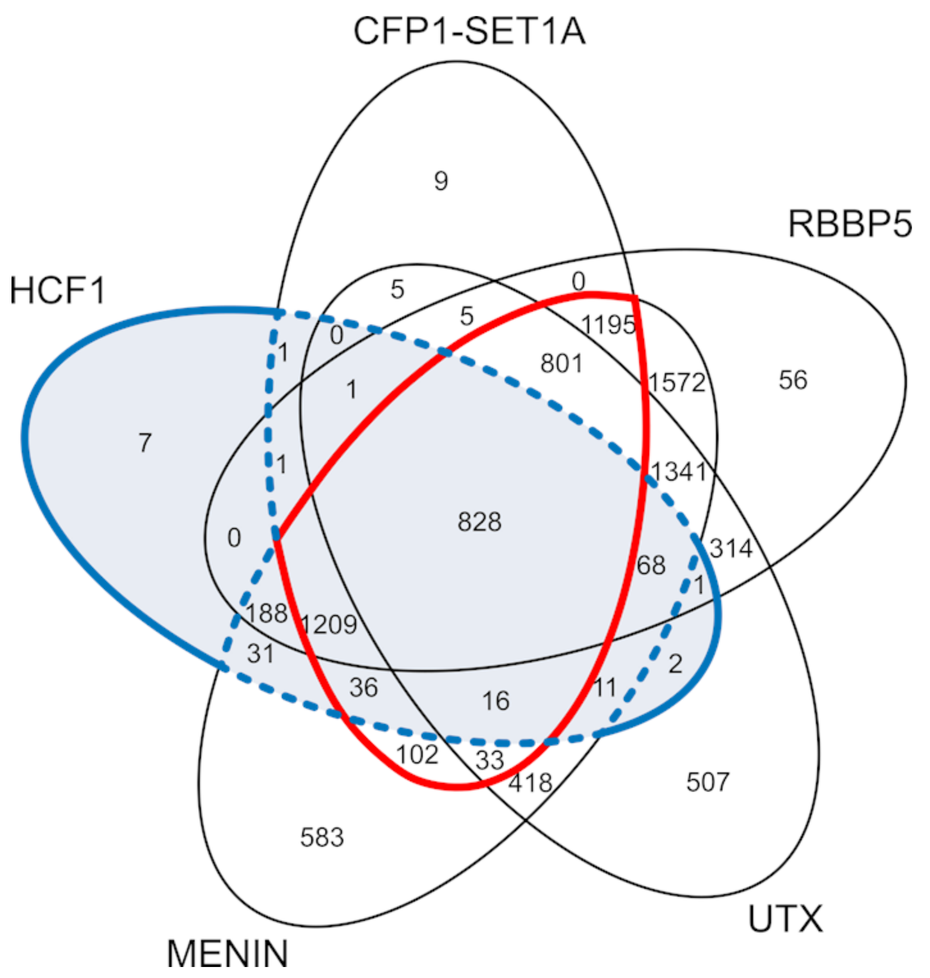

Fig. S7 – Overlap of TrxG subunit ChIP peaks in a high-confidence subset of regions. SET1A complexes are represented by CFP1-SET1A colocalisation. MLL1/2 complexes are represented by Menin, and MLL3/4 complexes are represented by UTX, respectively. HCF1 is found in SET1A/B and MLL1/2 complexes, and RBBP5 is a member of SET1A/B and MLL1/2/3/4 complexes. Red outline (4220 peaks) shows strong colocalisation of Menin and CFP1-SET1A, accounting for the vast majority (99.5%) of 4242 CFP1-SET1A and half (50.0%) of 8432 Menin peak regions. Majority (87.0%, 2089/2400 peaks) of HCF1 (blue region) is accounted for by approximately half (49.5%, 2089/4220) of regions of Menin-SET1A-CFP1 colocalisation. Regions where either SET1A-CFP1 or Menin or both are colocalised with HCF1 (blue dashed line) accounts for nearly all (99.6%, 2390/2400) HCF1 regions, suggesting that HCF1 bound to DNA is primarily present as part of SET1A/B or MLL1/2 complexes.

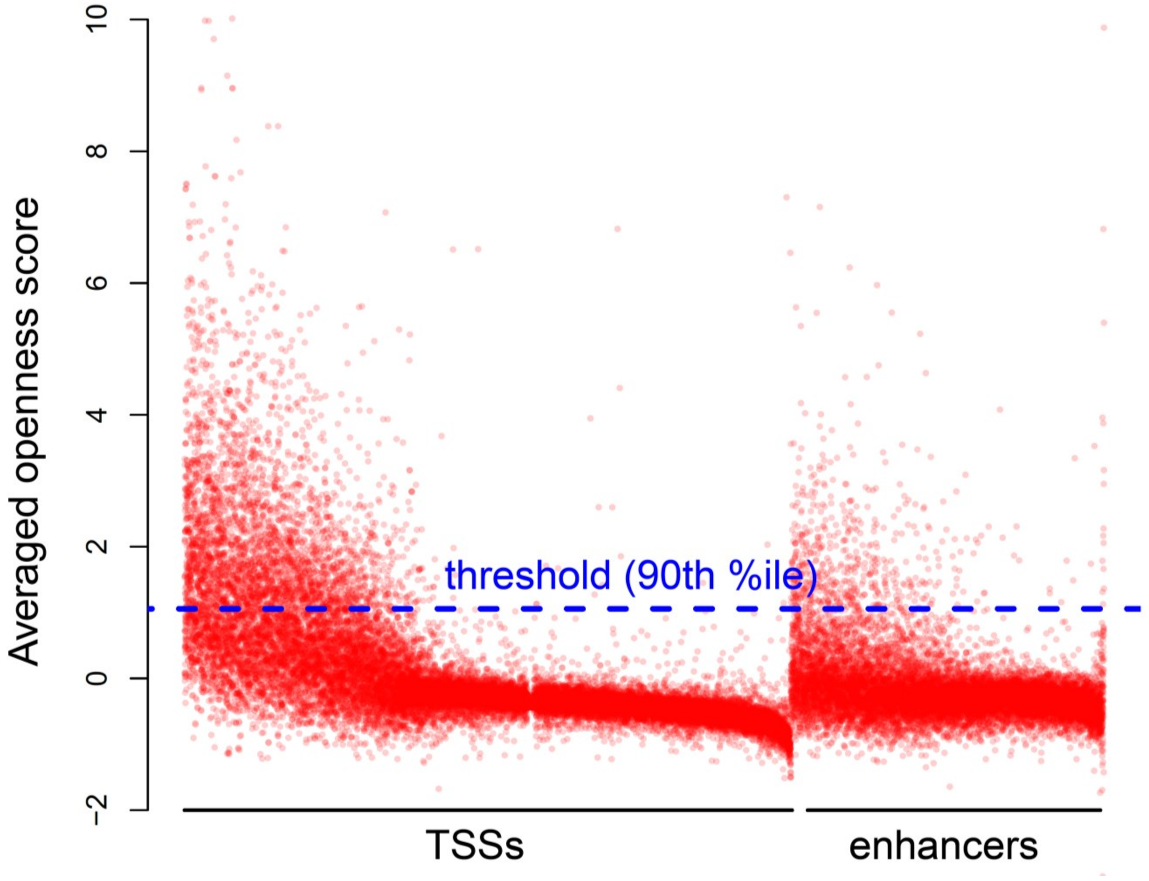

Fig. S8 – Chromatin accessibility in TSSs and enhancers in erythroid cells as measured by ATAC-seq and DNase-seq. 1x-normalised, input-subtracted signals from ATAC-seq and DNase were averaged in a 2kb window about TSSs and putative enhancers. Z-score transformed values for ATAC-seq and DNase-seq at a given locus were averaged.

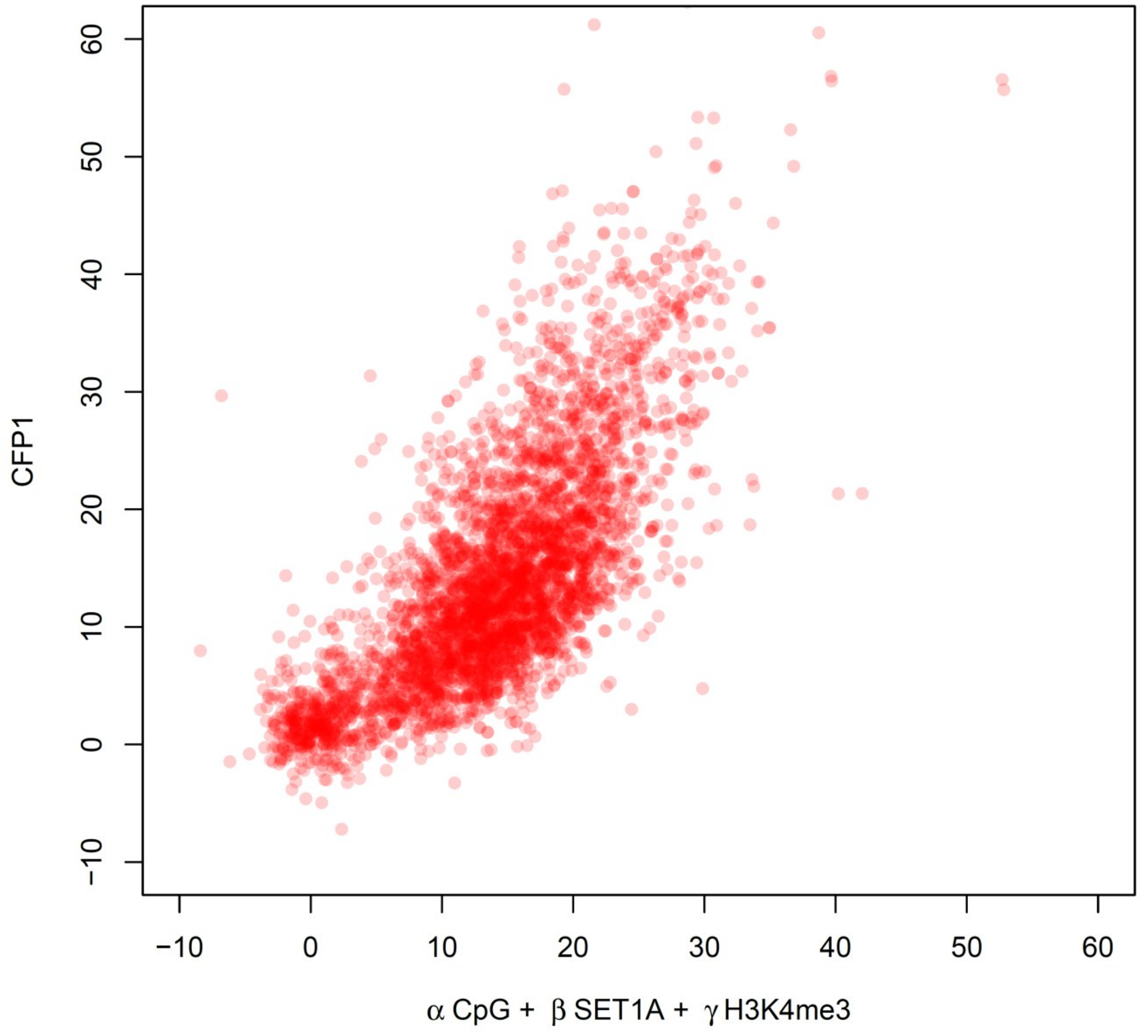

Fig. S9 – Relationship of CFP1 signal to three predictive factors in top-decile open chromatin regions. A linear combination of CpG density and SET1A and H3K4me3 ChIP signals explains a substantial fraction of variation in CFP1 ChIP signal.

Table S1 – Bias of CFP1 for CGI TSSs in cell types and gene classes

Cell type	Gene class	Odds ratio (95% CI)	p-value (Fisher's exact test)
Erythroid	tissue-specific	7.5 (4.1, 13.8)	1.6×10^{-12}
	housekeeping	9.1 (7.0, 11.7)	1.3×10^{-55}
Lymphoid (EBV)	tissue-specific	23.1 (9.3, 66.2)	1.3×10^{-16}
	housekeeping	5.9 (4.1, 8.6)	2.7×10^{-19}

Table S2 – Bias of CFP1 for housekeeping gene TSSs

Cell type	TSS class	Odds ratio (95% CI)	p-value (Fisher's exact test)
Erythroid	CGI	2.5 (1.7, 3.6)	1.2×10^{-5}
	Non-CGI	2.1 (1.2, 3.5)	4.7×10^{-3}
Lymphoid (EBV)	CGI	2.0 (1.4, 3.0)	3.8×10^{-4}
	Non-CGI	8.0 (3.2, 22.8)	2.3×10^{-7}

Table S3 – Motifs associated with CFP1 peaks.*

	Motif	Statistics**: EBV %, ERY %, background %, opt p-value*	Best motif match	Name/JASPAR ID (homer similarity score)
non-CGI				
1	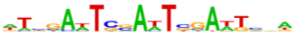	11.2%, 0.0%, 0.0%, 1.6×10^{-263}	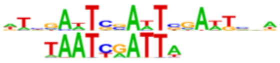	PAX3/MA0780.1 (0.54)
2	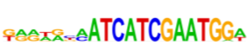	11.1%, 0.0%, 0.0%, 9.4×10^{-246}	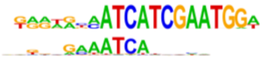	Hdx/PH0037.1 (0.56)
3	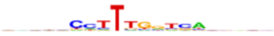	8.9%, 3.5%, 0.5%, 2.6×10^{-53}	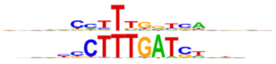	Lef1_1/PB0040.1 (0.77)
4	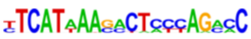	0.3%, 1.7%, 0.0%, 2.5×10^{-51}	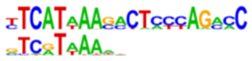	HOXC10/MA0905.1 (0.55)
5	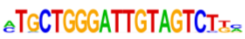	0.1%, 1.8%, 0.0%, 1.4×10^{-50}	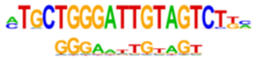	GFY/(Homer db) (0.62)
CGI				
1	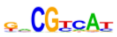	19.1%, 53.9%, 9.7%, 1.8×10^{-102}	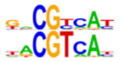	Atf1/MA0604.1 (0.89)
2	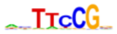	33.3%, 63.1%, 16.7%, 1.8×10^{-79}	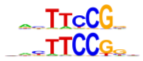	Elk1/(Homer db) (0.97)
3	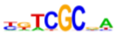	44.9%, 82.8%, 31.6%, 4.0×10^{-43}	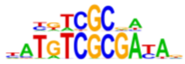	ZBED1/MA0749.1 (0.80)
4	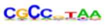	16.1%, 39.7%, 9.1%, 1.2×10^{-35}	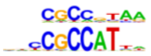	YY2/MA0748.1 (0.71)
5	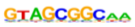	52.0%, 65.4%, 28.7%, 6.8×10^{-31}	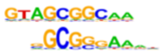	E2F4/(Homer db) (0.64)

* peaks were found using the spp library in R.

** statistics represent fraction of CFP1 peaks with the motif in ERY or EBV, relative to background sequences matched for sequence content in 3-base oligos. Peaks in ERY and EBV were the subset of the 1000 highest peaks unique to that cell type after removal of peaks found in both cell types. In ERY, these cell type-specific peaks consisted of 635 and 709 peaks with and without CGIs. In EBV, these cell type-specific peaks consisted of 633 and 722 peaks with and without CGIs.

Table S4 – Dependence* of CFP1 ChIP signal in erythroid cells on covariates putatively associated with its binding.

Covariate	raw Pearson correlation across all TSSs and putative enhancers (% variance explained)	correlation in top-decile open† regions (% variance explained)
CpG density	**0.445 (19.8)**	**0.390 (15.2)**
SET1A	**0.439 (19.3)**	**0.400 (16.0)**
HCF1	**0.338 (11.4)**	0.179 (3.2)
RBBP5	**0.665 (44.3)**	0.246 (6.1)
H3K4me1	-0.077 (0.6)	-0.073 (0.5)
H3K4me3	**0.759 (57.6)**	**0.534 (28.6)**
Chromatin accessibility†	**0.628 (39.4)**	0.162 (2.6)

Note, covariates accounting for more than 10% of variance are shown in bold.

* Values shown are Pearson product moment correlations *r*, with percentage variance of CFP1 signal explained shown in parentheses. Correlations shown are between signals normalised to 1x coverage, with input subtracted, and averaged in a 2kb window around TSSs and putative enhancers.

† Chromatin accessibility is measured using ATAC-seq and DNase-seq signals. 1x-normalised, input-subtracted signals from ATAC-seq and DNase were averaged in a 2kb window about TSSs and putative enhancers. Z-score transformed values for ATAC-seq and DNase-seq were averaged.

Table S5 – Analysis of variance of CFP1 signal in top-decile open-chromatin* regions surrounding TSSs and putative enhancers.

Covariate	dof	sum-squared	mean-squared	F(1,4113)	P-value
CpG density	1	74275	74275	1196	2.4e-230
SET1A	1	108673	108673	1750	4.7e-319
H3K4me3	1	50478	49871	803	1.4e-161
Residuals	4113	255354	62		

* Chromatin accessibility is measured using ATAC-seq and DNase-seq signals. 1x-normalised, input-subtracted signals from ATAC-seq and DNase were averaged in a 2kb window about TSSs and putative enhancers. Z-score transformed values for ATAC-seq and DNase-seq were averaged.
